# Evidence that the Great Pacific Garbage Patch is rapidly accumulating plastic

**DOI:** 10.1038/s41598-018-22939-w

**Published:** 2018-03-22

**Authors:** L. Lebreton, B. Slat, F. Ferrari, B. Sainte-Rose, J. Aitken, R. Marthouse, S. Hajbane, S. Cunsolo, A. Schwarz, A. Levivier, K. Noble, P. Debeljak, H. Maral, R. Schoeneich-Argent, R. Brambini, J. Reisser

**Affiliations:** 1 The Ocean Cleanup Foundation, Martinus Nijhofflaan 2, Delft, 2624 ES The Netherlands; 2 The Modelling House, 66b Upper Wainui Road, Raglan, 3297 New Zealand; 3 Teledyne Optech, Inc., 7225 Stennis Airport Road, Kiln, MS 39556 USA; 4 School of Civil Engineering and Surveying, Faculty of Technology, University of Portsmouth, Portland Building, Portland Street, Portsmouth, PO1 3AH UK; 5 Department of Biology, Marine Biology and Environmental Science, Roger Williams University, 1 Old Ferry Road, Bristol, RI 02809 USA; 6 Sorbonne Universités, UPMC Univ Paris 06, CNRS, Laboratoire d’Océanographie Microbienne (LOMIC), Observatoire Océanologique, F-66650 Banyuls/mer, France; 7 Department of Civil, Geo and Environmental Engineering, Technical University Munich, Arcisstraße 21, Munich, 80333 Germany; 8 ICBM-Terramare, Carl von Ossietzky University Oldenburg, Schleusenstr. 1, Wilhelmshaven, 26382 Germany; 9 Civil Engineering Department, Aalborg University, Fredrik Bajers Vei 5, Aalborg, 9100 Denmark

## Abstract

Ocean plastic can persist in sea surface waters, eventually accumulating in remote areas of the world’s oceans. Here we characterise and quantify a major ocean plastic accumulation zone formed in subtropical waters between California and Hawaii: The Great Pacific Garbage Patch (GPGP). Our model, calibrated with data from multi-vessel and aircraft surveys, predicted at least 79 (45–129) thousand tonnes of ocean plastic are floating inside an area of 1.6 million km^2^; a figure four to sixteen times higher than previously reported. We explain this difference through the use of more robust methods to quantify larger debris. Over three-quarters of the GPGP mass was carried by debris larger than 5 cm and at least 46% was comprised of fishing nets. Microplastics accounted for 8% of the total mass but 94% of the estimated 1.8 (1.1–3.6) trillion pieces floating in the area. Plastic collected during our study has specific characteristics such as small surface-to-volume ratio, indicating that only certain types of debris have the capacity to persist and accumulate at the surface of the GPGP. Finally, our results suggest that ocean plastic pollution within the GPGP is increasing exponentially and at a faster rate than in surrounding waters.

## Introduction

Global annual plastic consumption has now reached over 320 million tonnes with more plastic produced in the last decade than ever before^1^. A significant amount of the produced material serves an ephemeral purpose and is rapidly converted into waste. A small portion may be recycled or incinerated while the majority will either be discarded into landfill or littered into natural environments, including the world’s oceans^2^. While the introduction of synthetic fibres in fishing and aquaculture gear represented an important technological advance specifically for its persistence in the marine environment, accidental and deliberate gear losses became a major source of ocean plastic pollution^3^. Lost or discarded fishing nets known as ghostnets are of particular concern as they yield direct negative impacts on the economy^4–7^ and marine habitats worldwide^8,9^.

Around 60% of the plastic produced is less dense than seawater^10^. When introduced into the marine environment, buoyant plastic can be transported by surface currents and winds^11^, recaptured by coastlines^12,13^, degraded into smaller pieces^14^ by the action of sun, temperature variations, waves and marine life^10^, or lose buoyancy and sink^15^. A portion of these buoyant plastics however, is transported offshore and enters oceanic gyres^16^. A considerable accumulation zone for buoyant plastic was identified in the eastern part of the North Pacific Subtropical Gyre^17^. This area has been described as ‘a gyre within a gyre’^18^ and commonly referred to as the ‘Great Pacific Garbage Patch’ (GPGP^19,20^). The relatively high concentrations of ocean plastic occurring in this region^21,22^ are mostly attributed to a connection to substantial ocean plastic sources in Asia^23,24^ through the Kuroshio Extension (KE) current system^25^ as well as intensified fishing activity in the Pacific Ocean^26^.

Most available data on quantities and characteristics of buoyant ocean plastic are derived from samples collected with small sea surface trawls initially developed to collect neustonic plankton^27^. Due to their small aperture (0.5–1 m width, 0.15–1 m depth) and limited surface area covered, they could underestimate loads of rarer and larger plastic objects such as bottles, buoys and fishing nets. In an attempt to overcome this misrepresentation, a research team^21^ combined net tow data with information from vessel-based visual sighting surveys. They found that while small, millimetre-sized pieces (<4.75 mm) count in trillions at global scale, they only represent a small mass portion (13%) of the total available buoyant material. Nevertheless, vessel-based sightings data yielded high uncertainties due to differences in survey protocols across research groups and difficulties in estimating the mass of sighted objects. Historical datasets on buoyant ocean plastic are also sparse in space and time^28^. To circumvent such limitations, recent studies have coupled datasets^21,22,29^ with dispersal models^30–32^ to predict ocean plastic pollution levels worldwide. Outputs from ocean plastic transport model are generally integrated over several years and calibrated against datasets collected during different seasons, years and decades. However, such method may misrepresent ocean plastic transportation and accumulation as these processes are closely associated with seasonal and inter-annual variability^18,25^.

In this study, we characterized and quantified buoyant ocean plastics inside the GPGP. Between July and September 2015, we conducted a multi-vessel expedition to collect surface trawl samples within and around the GPGP region and obtain a representative distribution of buoyant plastic concentrations in this region. In October 2016, we conducted an aerial survey to obtain geo-referenced imagery that sampled greater sea surface area and improved estimations for debris larger than 0.5 m. Our final dataset, containing measured concentrations for ocean plastic of various sizes and types, was used to calibrate a multi-source and multi-forcing ocean plastic transport model. We calibrated our numerical model using monthly averages of predicted concentrations that reflected seasonal and inter-annual changes of the GPGP position. As such, this study is a first attempt at introducing a time-coherent dynamic model of floating debris accumulation in the GPGP. This allowed us to compare our findings with historical observations (1970s to present) and assess the long-term evolution of ocean plastic concentrations within and around the GPGP.

## Methods

### Sampling

**Figure 1:**
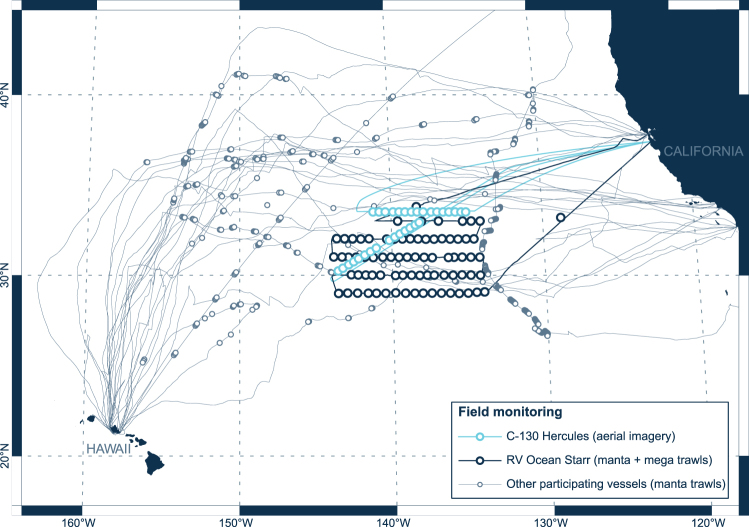
Field monitoring effort. Vessel (grey and dark blue lines) and aircraft (light blue lines) tracks and locations where data on buoyant ocean plastic concentrations were collected (circles). Grey circles (*n* = 350) represent areas sampled with a single Manta net tow by 17 participating vessels, between July and September 2015. Dark blue circles (*n* = 76) represent areas sampled with paired Manta and paired Mega net tows by *RV Ocean Starr*, between July and August 2015. Light blue circles (*n* = 31) show locations of RGB geo-referenced mosaics collected from a *C-130 Hercules* aircraft, in October 2016. This map was created using QGIS version 2.18.1 (www.qgis.org).

From July 27^th^ to September 19^th^ 2015, a total of 652 surface net tows were carried out between 25°N–41°N and 129–156°W by 18 participating vessels. In October 2016, we revisited our study area by conducting two flights with a Hercules C-130 aircraft that collected aerial imagery (*n* = 7,298 single-frame mosaics) to better quantify the larger and rarer >50 cm plastic objects (Fig. 1).

Vessels carried out net tows of 0.35–4 hours duration, while navigating at 0.7–6.8 knots. All trawls were designed to move away from the vessel to avoid wake effects on the capture efficiency of the devices. All vessel crews were trained with online material and one-to-one workshops that had been conducted prior to departure. While towing the trawl, the most experienced sailor aboard the vessel estimated the sea state (Beaufort scale) by measuring wind speeds and observing wave heights. This data was recorded in the standard datasheets provided, alongside the date, duration, as well as initial and final coordinates of each tow. The location and length of all net tows were confirmed during the post-processing phase by inspecting the position data from GPS trackers installed on all participating vessels. Most sampling stations encompassed a single net tow (*n* = 350 sampling stations) using a Manta trawl (0.5 mm square mesh, 90 cm × 15 cm mouth), which is one of the standard devices for quantifying plastic pollution levels. With the largest participating vessel (*RV Ocean Starr*), we simultaneously towed two Manta trawls, alongside two large Neuston trawls (1.5 cm square mesh, 6 m × 1.5 m mouth, of which 0.5 m above the water line; thereafter called ‘Mega trawls’) at every sampling location (*n* = 76 stations). After each Manta net tow, the net was rinsed from the outside with seawater, and its single-use cod-end removed, closed with staples and placed in an individual zip-lock bag. After each Mega trawl tow, the net was also rinsed from the outside with seawater and its large cod-end opened in a box filled with seawater. All buoyant plastics were then removed, wrapped in aluminium and placed in labelled plastic bags. The whole content captured by the Manta trawls was stored, while the organisms captured by the Mega trawls (mostly alive) were released back into the ocean. All samples were stored in a fridge or freezer while at-sea, and in a FedEx cool box (2–8 °C) or reefer (−2 °C) while being shipped to the laboratory. Even though we were careful when handling samples, some debris items may have been broken during transportation, leading to some bias in our debris size distribution. Detailed information related to these net tows (i.e. coordinates, metocean conditions, sampling times and durations) is provided in Figshare^33^.

The aerial surveys sampled a far greater area (311.0 km^2^) than the trawl surveys described above (3.9 km^2^ and 13.6 km^2^, for Manta and Mega net tows, respectively), thus yielding a more reliable quantification of debris larger than 50 cm, which are relatively rare. Both flights started and ended at Moffett Airfield near Mountain View, California. The first aerial survey was conducted on October 2^nd^ 2016 sampling from 18:56 to 21:14 UTC time, at a constant latitude of 33.5°N, and longitudes varying from 141.4°W to 134.9°W. The second survey started on October 6^th^ 2016 sampling from 22:14 to 0:37 UTC, from 30.1°N, 143.7°W to 32.9°N, 138.1°W. While in survey mode, the aircraft flew at an altitude of approximately 400 m and at a ground speed of 140 knots. Sampling transects targeted areas where the sea state conditions were the lowest, based on the weather forecast, including sea surface atmospheric pressure, cloud cover, wind speed at 10 m above sea level and boundary surface layer height provided by NOAA’s Global Forecasting System, as well as significant wave height and peak period data distributed by NOAA’s WaveWatch3 model outputs. Even though we surveyed floating debris using trained observers and three types of sensor (Lidar, SWIR imager, and RGB camera), here we only analyse information coming from the geo-referenced mosaics produced by a RGB camera (CS-4800i) that generally took photographs every second during surveying time (frame size = ~360 m across track, ~240 m along track, ~0.1 m resolution).

### Trawl samples processing

Trawl samples were separately washed into a sieve tower (five Glenammer Engineering Ltd sieves, with 0.05 cm, 0.15 cm, 0.5 cm, 1.5 cm, and 5 cm square apertures) that divided the material into the following size classes: 0.05–0.15 cm, 0.15–0.5 cm, 0.5–1.5 cm, 1.5–5 cm, and >5 cm. Debris items >5 cm were then manually sorted into 5–10 cm, 10–50 cm, and >50 cm classes by measuring the object lengths (widest dimension of the object) with a ruler. Buoyant debris was separated from biomass by placing the material within each sieve in filtered saltwater (salinity 3.5%, temperature 19–23 °C). Lab personnel stirred the material many times to insure floating particles were detached from the biomass material. Floating objects identified as buoyant debris were manually extracted from the water surface using forceps, separated into types, and counted. Buoyant debris was classified into material type (plastic, glass, paraffin, tar, rubber, wood, pumice, seed or unknown), with plastics being further divided into the following categories: (1) ‘H’ type – fragments and objects made of hard plastic, plastic sheet or film; (2) ‘N’ type – plastic lines, ropes, and fishing nets; (3) ‘P’ type – pre-production plastic pellets in the shape of a cylinder, disk or sphere; and (4) ‘F’ type – fragments or objects made of foamed material (e.g. expanded polystyrene). Once counted and categorized, the pieces were washed with distilled water, transferred to aluminium dishes, dried overnight at 60 °C, and weighed using an OHAUS Explorer EX324M (0.0001 g readability) for objects <5 cm, and a OHAUS Explorer EX12001M (0.1 g readability) for objects >5 cm.

To best characterize the ocean plastic accumulating within the GPGP, we performed additional analyses with the material collected. Firstly, 10 pieces within each plastic size/type category (*n* = 220 pieces) were selected for polymer composition analysis by Fourier-transform infrared spectroscopy (FT-IR). The readings were done using a Perkin Elmer Spectrum 100 FT-IR equipped with a universal ATR accessory (range = 600–4000 cm^−1^). The respective polymer type was determined by comparing sample FT-IR spectra against known spectra from a database (Perkin-Elmer ATR of Polymers Library). Secondly, we screened all plastic debris collected for production dates, as well as any writings statements giving information on its origin (i.e. language and ‘made in’ statements). Lastly, we classified plastic items from ‘H’ and ‘L’ types collected at 30 *RV Ocean Starr* stations into object types (e.g. bottle lids, bags, bottles, etc). As ‘H’ objects larger than 50 cm were relatively rare, we analysed 10 extra *RV Ocean Starr* stations for this type/size category. If the object type of a fragment could not be determined, we classified the piece as either hard plastic fragment or film fragment depending on its wall thickness and flexibility^34^. We used Manta trawl samples to characterise objects within size classes 0.15–0.5 cm, 0.5–1.5 cm, and 1.5–5 cm, and Mega trawl samples to characterise objects within size classes 5–10 cm, 10–50 cm, and >50 cm. Plastics within our smallest size class (0.05–0.15 cm) were not considered in this ‘Object Type’ analysis due to the difficulty of handling and identifying small fragments.

The numerical/mass concentrations of buoyant plastic items (count/kg of plastic per km^2^ of sea surface) measured by each net tow were calculated for all plastic size/type categories separately. To do so, we divided the count and weight of plastic objects within each category by the towed area of the sample. We calculated the towed area by multiplying net mouth width (90 cm for Manta trawl, 6 m for Mega trawl) by tow length (determined from GPS position data). The average area covered by Manta net tows was 0.008 km^2^ (SD = 0.004, min–max: 0.001–0.018 km^2^), while the average area covered by Mega net tows was 0.090 km^2^, (SD = 0.013, min–max: 0.046–0.125 km^2^). As buoyant plastics can be missed by surface trawls due to wind-driven mixing, we then estimated the ‘depth-integrated’ mass and numerical plastic concentrations (*Ci*) for all type/size categories at each of the trawl sampling locations using the equations described in ref.^35^. Supplementary Methods 1 provides details on how *Ci* was calculated as a function of ocean plastic terminal rising velocity (*Wb*), depth sampled by the trawl, and sea state. It also describes how we measured *Wb* for each of the type/size categories of this study. After comparing plastic concentration results obtained by paired Manta and Mega net tows (*n* = 76 locations), we decided to use Manta and Mega trawl samples to quantify debris 0.05–5 cm and 5–50 cm in size, respectively. The comparison results and reasoning behind such decision in provided in Supplementary Methods 2.

### Aerial imagery processing

All RGB images taken during our survey flights (*n* = 7,298) were georeferenced using accurate aircraft position and altitude data collected during the surveys. They were then inspected by two trained observers and a detection algorithm. Observers inspected all images at full-screen on a Samsung HD monitor (LU28E590DS/XY) and those single-frame mosaics containing debris were uploaded into QGIS software (Version 2.18.3–Las Palmas) to record their position and characteristics. We trust we had a very small number of false positives and a high number of false negatives. This is because the observers took a conservative approach: they only logged features as debris when they were very confident with its identification. As such, many features that could be debris, but resembled other natural features, such as sun glint and breaking wave, were not logged into our ocean plastic dataset. Once this work was finalised, we ran an experimental algorithm capable of detecting potential debris in all our RGB mosaics as a quality control step. To avoid any false positives, all features detected by the algorithm were also visually inspected by an observer and only those visually identified as debris were logged in our QGIS database. For every sighting, we recorded position (latitude, longitude), length (widest dimension of the object), width, and object type: (1) ‘bundled net’ – a group of fishing nets bundled tightly together; they are commonly colourful and of a rounded shape; (2) ‘loose net’ – a single fishing net; they were generally quite translucent and rectangular in shape; (3) ‘container’ – rectangular and bright objects, such as fishing crates and drums; (4) ‘rope’ – long cylindrical objects around 15 cm thick; (5) ‘buoy/lid’, rounded bright objects that could be either a large lid or a buoy; (6) ‘unknown’ – objects that are clearly debris but whose object type was not identified, they were mostly irregular-shaped items resembling plastic fragments; and (7) Other – only one object was successfully identified but did not belong to any category above: a life ring. We recorded 1,595 debris items (403 and 1,192 in flights 1 and 2 respectively); 626 were 10–50 cm and 969 were >50 cm in length. Most of them were classified as ‘unknown’ (78% for 10–50 cm, 32% for >50 cm), followed by ‘buoy or lid’ (20%) and ‘bundled net’ (1%) for 10–50 cm debris, and by ‘bundled net’ (29%), ‘container’ (18%), ‘buoy or lid’ (9%), ‘rope’ (6%), and ‘lose net’ (4%) for >50 cm debris. To calculate ocean plastic concentrations, we grouped the geo-referenced images into 31 ~10 km^2^ mosaics. For numerical concentrations, we simply divided the number of debris pieces 10–50 cm and >50 cm within each mosaic by the area covered. To estimate mass concentrations, we had to first estimate the mass of each object spotted, then we separately summed the mass of 10–50 cm and >50 cm debris within each mosaic by the area covered. More information on how we estimated the mass of each spotted objects is provided in Supplementary Methods 3.

### Numerical model formulation

Ocean plastic pathways can be represented by Lagrangian particle trajectories^31^. In our framework, particles were advected by the following environmental drivers: sea surface currents, wave induced Stokes drift and winds. Starting from identical particle releases, we produced a series of forcing scenarios to represent the diversity in shape and composition of ocean plastics. Starting from using sea surface current only, we gradually added forcing terms representing the actions of atmospheric drag and wind waves on buoyant debris. The action of wind was simulated by considering the displacement of particles as a fraction of wind speed at 10 m above sea level. This is referred as the ‘windage coefficient’. We assessed different windage coefficient scenarios including 0%, 0.1%, 0.5%, 1%, 2% and 3%. We sourced global sea surface currents (1993 to 2012) from the HYCOM + NCODA global 1/12° reanalysis (experiment 19.0 and 19.1^36–38^), and wind (10 m above sea level) speed and direction data (1993 to 2012) from NCEP/NCAR global reanalysis^39^. Wave induced Stokes drift amplitude was calculated using wave spectrum bulk coefficients (significant wave height, peak wave period and direction) from Wavewatch3 model outputs^40^.

For every forcing scenario, particles were identically and continuously released in time from 1993 to 2012 following spatial distributions and amplitudes of significant ocean plastic sources on land (coastal population hotspots^23^ and major rivers^24^) as well as at sea (fishing^26,41^, aquaculture^42^ and shipping industries^43^). Source scenarios were combined using relative source contribution as well as geographical distribution presented in Supplementary Methods 4. We advected global particles in time using the forcing scenarios described above and successfully reproduced the formation of oceanic garbage patches, with the shape and gradient of particle concentrations in these areas differing amongst forcing scenarios. We computed daily particle visits over 0.2° resolution grids corresponding to our observation domain and extending from 160°W to 120°W in longitude and 20°N to 45°N in latitude. The number of daily particle visits was uniformized over the total number of particles present in the global model at a given time. The model-predicted non-dimensional concentration *δ*_*i*_ of cell *i*, was calculated as follows:

1 $${\delta }_{i}=\sum _{s}{\alpha }_{s}{\delta }_{i,s}$$

where α_s_ is the non-dimensional weight relative to the contribution of source *s* and *δ*_*i*,*s*_ is the percentage of global particles from source *s* in cell *i*. *δ*_*i*,*s*_ is calculated with the number of particles *n*_*i*,*s*_ from source *s* in cell *i* over the total number of global particles Σ_*i*_*n*_*s*_ from source *s*:

2 $${\delta }_{i,s}=\frac{{n}_{i,s}}{{\sum }_{i}{n}_{s}}$$

### Numerical model calibration

We collected measurements at sea in 2015 and 2016, but our numerical model uses ocean circulation reanalysis covering the period from 1993 to 2012. Modelled ocean circulation data post-2012 is available from HYCOM however not as a reanalysis product. As such, we decided not to use it in this study. As initial model particles released in 1993 significantly start to accumulate in the area after about 7 years, we averaged uniformized daily particle visits over 12 years, from 2000 to 2012. We grouped observed debris size classes in four categories: microplastics (0.05–0.5 cm), mesoplastics (0.5–5 cm), macroplastics (5–50 cm) and megaplastics (>50 cm). We compared model predictions against depth-integrated microplastic concentrations as this dataset collected by Manta trawls had the largest spatial coverage. Mass concentrations derived from trawl measurements were grouped in 0.2-degree resolution cells and compared against model-predicted non-dimensional concentration δ for the five different forcing scenarios. The best model fit was found for the forcing scenario with sea-surface current only (*R*^2^ = 0.52, *n* = 277 cells). The regression coefficient declined as we increased the atmospheric drag term (*R*^2^ = 0.39 to 0.21 depending on windage coefficient).

As we analysed the accumulation of model particles in the GPGP region, we noticed significant seasonal and inter-annual variations of the GPGP position. The modelled GPGP dimensions was relatively consistent throughout our 12 years of analysis, but the relative position of this accumulation zone varied with years and seasons. We first decided to test our model for seasonal variation by comparing our microplastic concentrations (measured in July – September 2015) against modelled concentrations averages for the July–September periods of 2000 to 2012. This comparison yielded poorer results (*R*^2^ = 0.46 to 0.21, depending on forcing scenario) than with the 12 years average solution (R^2^ = 0.52) as the July–September GPGP position varied substantially among years.

The relation between the accumulation of marine debris in the North Pacific and climate events such as El Niño Southern Oscillation (ENSO) and the Pacific Decadal Oscillation (PDO) has previously been discussed^18^. As such, to account for inter-annual variation, we compared latitudinal and longitudinal position of the GPGP against these two climate indexes: ENSO and PDO. We found that 2002 and 2004 were similar to the conditions experienced during our multi-vessel expedition. Thus, we compared our measurements against particle visit averages for July–September of 2002 and 2004 combined. This second attempt exhibited better results (*R*^2^ = 0.58 to 0.41, depending on forcing scenario), suggesting that climatic events such as ENSO or PDO influence the average position of the GPGP. Therefore, we decided to use the July–September average for 2002 and 2004, which better accounts for inter-annual variations in the GPGP position. More information on the selection of years for calibrating the model against trawl and aerial surveys data is provided in Supplementary Methods 5. The best fit between model predictions and microplastic observations was found once again for the forcing scenario with sea surface current only (*R*^2^ = 0.58, *n* = 277). The best regression fit between measured and modelled microplastic concentrations had *a* = −8.3068 and *b* = 0.6770 in the parametric formulation:

3 $$\,{c}_{mod}=\,{10}^{\frac{{\mathrm{log}}_{10}\delta -a}{b}}$$

**Figure 2:**
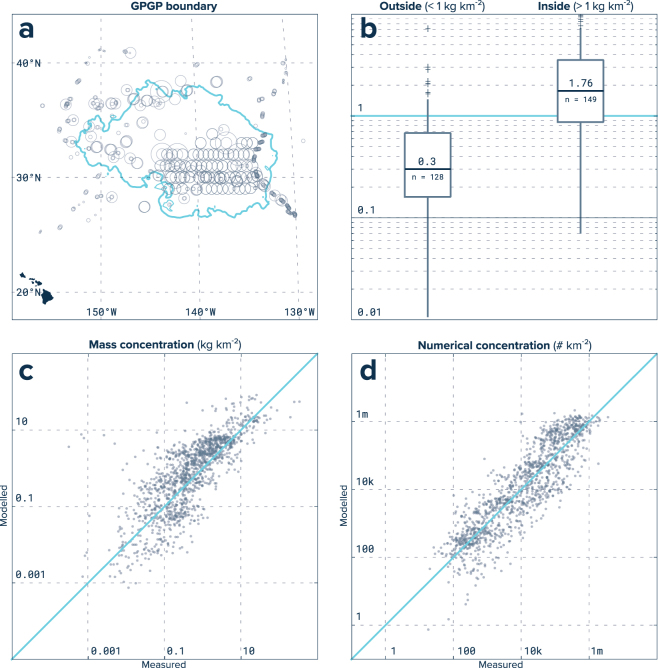
Numerical model calibration. (**a**) The GPGP boundary (blue line) is estimated by comparing microplastic concentration measurements (circles) to model particle visit averages that accounted for seasonal and inter-annual variations. This map was created using QGIS version 2.18.1 (www.qgis.org). (**b**) Model validation showing median measured mass concentration for microplastics of stations outside and inside our predicted 1 kg km^−2^ GPGP boundary. Bars extend from 25^th^ to 75^th^ percentile while whiskers extend to minimum and maximum non-outlier. Outliers are represented as crosses. (**c**) Measured mass concentrations versus modelled mass concentrations for microplastics, mesoplastics, macroplastics and megaplastics. (**d**) Same as (**c**) but with numerical concentrations.

From this formulation, we computed the modelled microplastics mass concentration in our domain area and extracted contour levels by order of magnitude, from 0.01 g km^−2^ to 10 kg km^−2^. The GPGP as defined in this study corresponds to the 1 kg km^−2^ microplastic mass concentration level covering an area of 1.6 million km^2^ and depicted as a bold line in Fig. 2a. As a validation, we categorized microplastics measurements inside and outside the 1 kg km^−2^ contour line (Fig. 2b). For stations inside the model-predicted GPGP, the median measured microplastic concentration was 1.8 kg km^−2^ (25^th^–75^th^ percentiles = 3.5–0.9 kg km^−2^) while for stations outside, the median was 0.3 kg km^−2^ (25^th^–75^th^ percentiles = 0.2–0.7 kg km^−2^). Using our calibrated microplastics distribution, we computed the mass and numerical concentration for individual size classes from scaling modelled concentrations by the ratio between average modelled microplastics distribution inside the GPGP and averaged measured concentrations per size class of stations inside the patch. A comparison between measured and modelled mass/numerical concentrations for all ocean plastic size classes is given in Fig. 2c and d.

Our confidence intervals were formulated to account for uncertainties in both sampling and modelling. For the trawl collection (i.e. micro-, meso- and macroplastics), we considered uncertainties related to the vertical-mixing corrections applied to surface concentrations using reported sea state and plastic’s rising velocities (see Supplementary Methods 1). For the aerial mosaics, we accounted for uncertainties related to estimating the mass of sighted objects based on correlations between top-view area and dry weight of objects collected in the trawls (see Supplementary Methods 3). Finally, to account for modelling uncertainties, we added (respectively subtracted) the standard error of measured concentration to (resp. from) the mean upper (resp. lower) mass concentration when scaling the microplastics distribution to individual size classes.

### Characterisation by types, sources and forcing scenarios

The total estimated mass load of ocean plastic in the GPGP by size classes were further divided by types. We calculated the average fraction in mass of individual ocean plastic types per sampling event for stations inside the patch (Supplementary Table 1) and derived the contribution of types ‘H’, ‘N’, ‘F’ and ‘P’. Further, as we predominantly observed debris originated from marine sources, we investigated the source contribution predicted by our calibrated modelled distribution. For individual model cells, we calculated the percentage of Lagrangian particle visits from individual sources. As initial particles were weighted in accordance to estimated global inputs, model particles from marine sources originally represented 28.1% of the total amount of material with fishing (17.9%), aquaculture (1.3%) and shipping (8.9%). We calculated the difference from this initial percentage value for each model cell and reported it to the predicted total mass concentration. In doing so, we defined ‘anomalies’ in marine source contribution in the North Pacific and expressed these in unit of mass per surface area. Finally, even though our calibrated model considered sea surface current only, we compared the predominance of forcing scenarios by evaluating the respective number of particle visits for each model cells. We computed contours around the GPGP for individual forcing scenarios in a way that the material contained inside each contour is equal to our initial forcing scenario (i.e. sea surface current only).

The dependence of the particle trajectory on the windage coefficient predicted by our model is in good agreement with sightings and modelling of debris originated from the 2011 Tohoku tsunami in Japan^44,45^. The first identified Japanese debris items that arrived after 10 to 12 months on the North American shores were objects with high windage such as buoys, boats and floating docks. Debris also arrived on Hawaiian Islands 18 months after the incident. The time of arrival was closely related to object types, starting in the first year with large oyster farm buoys and other floats, containers, and canisters. In the second year more buoys, tipped boats, fridges, and pallets arrived, followed later by timber beams and wooden debris. Our model predicted that only objects with a windage coefficient above 3% could arrive on Hawaii in the second year after the 2011 tsunami. Objects with windage coefficient ranging from 1 to 2% would reach Hawaii during the third year, while objects with no windage would mostly accumulate in the GPGP, north east of the archipelago.

### Long term analysis

The definition of a dynamic GPGP boundary that accounts for seasonal and inter-annual variabilities allowed us to estimate which sea surface trawl data points from the literature are inside or outside the GPGP region. Therefore, we used our calibrated model to assess the decadal evolution of microplastic mass concentrations (kg km^−2^) within and around the GPGP. Concentration data from the literature (Supplementary Table 2) was obtained from published datasets or digitized from figures when not available digitally^17,46,47^. When data was reported in unit of mass per volume of water^48^, we used the net tow depth to calculate the concentration per surface area unit. When only numerical concentration was reported^22,48^, we estimated mass concentration by using the average ocean plastic mass from net tows where both mass and numerical concentrations were reported (*m* = 3.53 mg, *SE*: 0.10 mg, *n* = 872).

We compared the model-predicted GPGP boundary with the locations of samples collected between 1999 and 2012^21,22,48,49^. Samples collected before 1999^17,46–48^ were compared against the GPGP position estimated for the sampled months and years in the 1999–2012 period that had similar ENSO and PDO values (See Supplementary Methods 6). Using our dynamic GPGP model boundary as reference, we classified each net tow into 3 categories: (1) sampled within the GPGP boundary, (2) sampled outside the GPGP boundary, but above 20°N and below 45°N and (3) sampled in the rest of the North Pacific. We only used net tows from the first two categories above so that concentration statistics for outside the patch were not biased by measurements taken in equatorial and polar waters, where concentrations were very low. We then grouped these microplastic concentration observations from plankton net trawls by decades, taking data recorded between 1965–1974 (*n* = 20 inside and *n* = 58 outside^17,48^), 1975–1984 (*n* = 0 inside and *n* = 19 outside^46^), 1985–1994 (*n* = 4 inside and *n* = 2 outside^47^), 1995–2004 (*n* = 2 inside and *n* = 252 outside^22,49^), 2005–2014 (*n* = 195 inside and *n* = 861 outside^21,22,48^) and finally 2015 (*n* = 288 inside and *n* = 213 outside; this study). We calculated the mean (± standard error) of measured microplastic mass concentration per decades for within and around the GPGP boundary. Finally, we extracted decadal trends by fitting an exponential function (*R*^2^ = 0.94) assuming null concentrations at the beginning of the 20^th^ century. The exponential fit exhibited better results than linear, quadratic or cubic functions (*R*^2^ = 0.71, *R*^2^ = 0.86 and *R*^2^ = 0.91, respectively).

## Results

### Ocean plastic loads and characteristics

**Figure 3:**
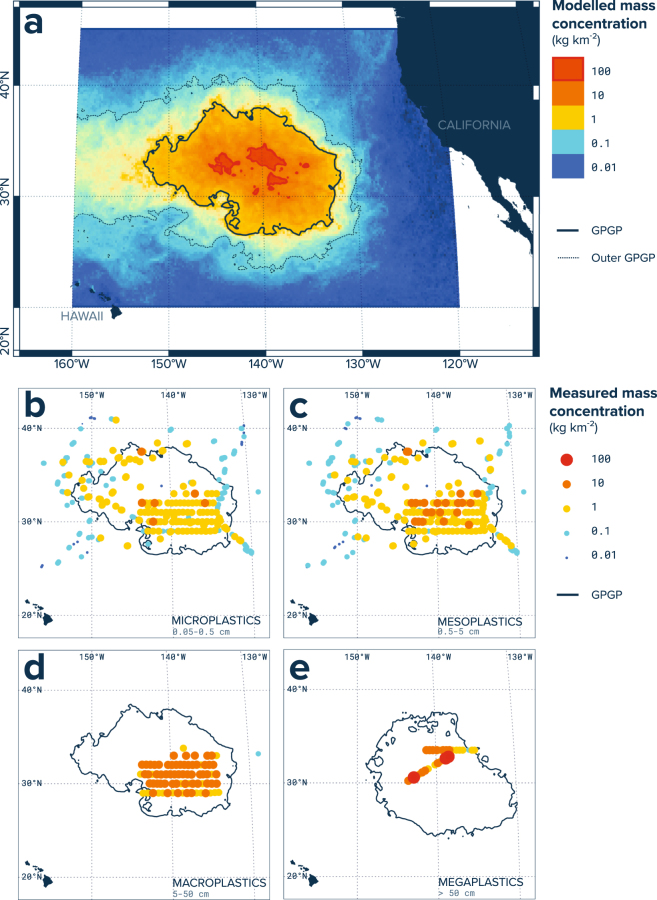
Modelled and measured mass concentration in the Great Pacific Garbage Patch (GPGP). (**a**) Ocean plastic mass concentrations for August 2015, as predicted by our data-calibrated model. The bold black line represents our established limit for the GPGP. (**b**) Microplastics (0.05–0.5 cm) mass concentrations as measured by Manta trawl (*n* = 501 net tows, 3.8 km^2^ surveyed). (**c**) Mesoplastics (0.5–5 cm) mass concentrations as measured by Manta trawl; d) Macroplastics (5–50 cm) mass concentrations as measured by Mega trawl (*n* = 151 net tows, 13.6 km^2^ surveyed); (**e**) Megaplastics (>50 cm) mass concentrations as estimated from aerial imagery (*n* = 31 mosaic segments, 311.0 km^2^ surveyed). All observational maps are showing mid-point mass concentration estimates as well as the predicted GPGP boundaries for the corresponding sampling period: August 2015 for net tow samples, and October 2016 for aerial mosaics. Maps were created using QGIS version 2.18.1 (www.qgis.org).

Plastics were by far the most dominant type of marine litter found, representing more than 99.9% of the 1,136,145 pieces and 668 kg of floating debris collected by our trawls. We estimated that an area of 1.6 million km^2^ holds ocean plastic concentrations ranging from 10 s to 100 s kg km^−2^ (Fig. 3). This area, which comprises ~87% of the ocean plastic material present in our model domain (120°W–160°W, 20°N–45°N), defines the Great Pacific Garbage Patch (GPGP) boundary for this study. We predicted that the GPGP contains a total of 1.8 (mid-point estimate, low: 1.1, high: 3.6) trillion plastic pieces weighing 79 k (45 k−129 k) tonnes, comprised of debris categorised in 4 size classes: microplastics (0.05–0.5 cm), mesoplastics (0.5–5 cm), macroplastics (5–50 cm), and megaplastics (>50 cm). Out of this total, we estimated 1.7 (1.1–3.5) trillion pieces and 6.4 k (4.1 k–12 k) tonnes of microplastics, 56 (39–104) billion pieces and 10 k (6.9 k–19 k) tonnes of mesoplastics, 821 (754–908) million pieces and 20k (18 k–22 k) tonnes of macroplastics, and 3.2 (2.7–3.6) million pieces and 42 k (16 k–75 k) tonnes of megaplastics (Table 1).

**Table 1:** Mass and numerical load per ocean plastic type and size within the 1.6 million km^2^ GPGP.

Size Class (cm)		Type H	Type N	Type P	Type F	All
0.05–0.15	(tonnes)	581 (351–1,252)	23 (14–49)	—	0.2 (0.1–0.4)	604 (365–1,302)
	(#)	1.0 10^12^ (6.3 10^11^–2.2 10^12^)	6.4 10^10^ (3.9 10^10^–1.4 10^11^)	—	4.5 10^8^ (2.7 10^8^–9.5 10^8^)	1.1 10^12^ (6.7 10^11^–2.3 10^12^)
0.15–0.5	(tonnes)	5,356 (3,498–10,035)	82 (54–154)	336 (219–630)	1.8 (1.2–3.4)	5,776 (3,772–10,821)
	(#)	5.6 10^11^ (3.6 10^11^–1.0 10^12^)	4.9 10^10^ (3.2 10^10^–9.0 10^10^)	2.2 10^10^ (1.5 10^10^–4.1 10^10^)	5.4 10^8^ (3.5 10^8^–9.9 10^8^)	6.3 10^11^ (4.1 10^11^–1.2 10^12^)
0.5–1.5	(tonnes)	4,703 (3,255–8,590)	159 (110–290)	0.9 (0.6–1.6)	3 (2–5)	4,865 (3,367–8,886)
	(#)	4.2 10^10^ (2.9 10^10^–7.8 10^10^)	9.5 10^9^ (6.6 10^9^–1.8 10^10^)	2.7 10^7^ (1.9 10^7^–5.0 10^7^)	3.9 10^7^ (2.7 10^7^–7.3 10^7^)	5.1 10^10^ (3.6 10^10^–9.6 10^10^)
1.5–5	(tonnes)	4,662 (3,220–9,642)	439 (303–907)	—	5 (4–11)	5,106 (3,527–10,560)
	(#)	2.8 10^9^ (2.0 10^9^–5.4 10^9^)	1.5 10^9^ (1.1 10^9^–2.8 10^9^)	—	1.5 10^7^ (1.1 10^7^–2.9 10^7^)	4.36 10^9^ (3.2 10^9^–8.3 10^9^)
5–10	(tonnes)	1,899 (1,714–2,166)	120 (108–137)	—	0.8 (0.7–0.9)	2,020 (1,823–2,304)
	(#)	2.1 10^8^ (1.9 10^8^–2.3 10^8^)	6.5 10^7^ (6.0 10^7^–7.3 10^7^)	—	4.7 10^5^ (4.3 10^5^–5.3 10^5^)	2.7 10^8^ (2.5 10^8^–3.1 10^8^)
10–50	(tonnes)	16,742 (15,265–18,330)	1,409 (1,284–1,542)	—	24 (22–27)	18,175 (16,572–19,899)
	(#)	3.4 10^8^ (3.1 10^8^–3.7 10^8^)	2.1 10^8^ (1.9 10^8^–2.3 10^8^)	—	9.0 10^5^ (8.2 10^5^–9.8 10^5^)	5.5 10^8^ (5.0 10^8^–6.0 10^8^)
>50	(tonnes)	3,217 (1,195–5,688)	39,144 (14,540–69,203)	—	—	42,362 (15,736–74,892)
	(#)	1.9 10^6^ (1.6 10^6^–2.2 10^6^)	1.3 10^6^ (1.1 10^6^–1.5 10^6^)	—	—	3.2 10^6^ (2.7 10^6^–3.6 10^6^)
All	(tonnes)	37,162 (28,500–55,704)	41,376 (16,414–72,283)	337 (220–631)	35 (29–46)	78,909 (45,163–128,665)
	(#)	1.6 10^12^ (1.0 10^12^–3.3 10^12^)	1.2 10^11^ (7.9 10^10^–2.5 10^11^)	2.2 10^10^ (1.5 10^10^–4.1 10^10^)	1.1 10^9^ (6.7 10^8^–2.1 10^9^)	1.8 10^12^ (1.1 10^12^–3.6 10^12^)

Plastic type H include pieces of hard plastic, plastic sheet and film, type N encompasses plastic lines, ropes and fishing nets, type P are pre-production plastic pellets, and type F are pieces made of foamed material.

**Figure 4:**
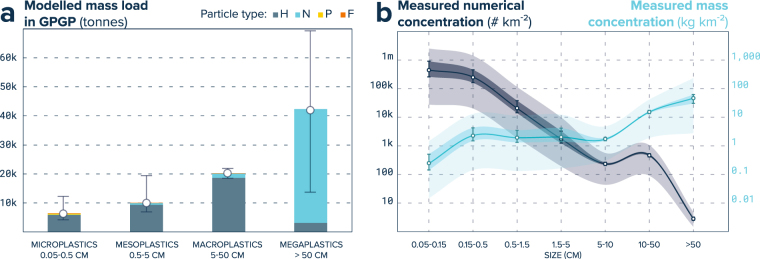
Ocean plastic size spectrum in the GPGP. (**a**) Plastic mass distribution within the GPGP between size (bars) and type (colours) classes. Plastic type H include pieces of hard plastic, plastic sheet and film, type N encompasses plastic lines, ropes and fishing nets, type P are pre-production plastic pellets, and type F are pieces made of foamed plastics. Whiskers extend from lower to upper estimates per size class, accounting for uncertainties in both monitoring and modelling methods. (**b**) Measured mass and numerical concentrations of GPGP ocean plastics. Dots represent the mean concentrations, the whiskers and darker shades represent our confidence intervals, and the lighter shades extend from the 5^th^ and 95^th^ percentile of measured concentrations.

More than three quarters of the GPGP plastic mass was contained in the upper size classes (>5 cm), with a respective total contribution of 25% and 53% for macroplastics and megaplastics (Fig. 4a). Plastic types ‘H’ (hard plastics, sheets and films) and ‘N’ (nets, ropes and lines) represented respectively 47% and 52% of the total GPGP plastic mass, with most of micro-, meso- and macroplastic mass coming from type ‘H’, and megaplastic from type ‘N’. Two additional plastic types, pellets (type ‘P’) and foams (type ‘F’) were also observed in a few size classes, but their overall contribution to the GPGP plastic load was minimal. For megaplastics, we could also assess the mass contributions of different object types. We estimated that 86% of their 42 k tonnes contribution was carried by fishing nets.

Megaplastics generally yielded the highest observed mass concentration with mean measured values of 46.3 kg km^−2^ (min–max: 0.4–428.1 kg km^−2^), followed by macroplastics with 16.8 kg km^−2^ (0.4–70.4 kg km^−2^), mesoplastics with 3.9 kg km^−2^ (0.0003–88.4 kg km^−2^), and microplastics with 2.5 kg km^−2^ (0.07–26.4 kg km^−2^). Regarding abundance however, microplastics and mesoplastics were by far the most numerous, with mean measured concentrations of 678,000 (min–max: 20,108–11,054,595) and 22,000 (261–321,712) pieces km^−2^ inside the GPGP against 690 (40–2,433) and 3.5 (0.5–11.6) pieces km^−2^ for macroplastics and megaplastics, respectively (Fig. 4b, Table 2).

**Table 2:** Mean observed mass and numerical concentrations within the 1.6 million km^2^ GPGP for different size and type of ocean plastics.

Size class	Type	Mean mass concentration (kg km^−2^)	Mean numerical concentration (# km^−2^)
Microplastic (0.05–0.5 cm)	H	2.33	643,930
	N	0.041	19,873
	P	0.13	14,362
	F	0.001	216
Mesoplastic (0.5–5 cm)	H	3.68	20,993
	N	0.23	803
	P	0.0003	3.6
	F	0.003	12
Macroplastic (5–50 cm)	H	15.53	640
	N	1.27	49
	F	0.021	0.7
Megaplastic (>50 cm)	H	3.52	0.3
	N	42.82	3.3
All	All	69.58	700,886

Plastic type H include pieces of hard plastic, plastic sheet and film, type N encompasses plastic lines, ropes and fishing nets, type P are pre-production plastic pellets, and type F are pieces made of foamed material.

The polymer composition of ocean plastic collected in the GPGP were analysed by Fourier-transform infrared spectroscopy. Polyethylene (PE) and polypropylene (PP) were by far the most common polymer types (Supplementary Table 3). Object type was rarely identifiable as most particles consisted of fragments. Plastic objects that could be identified (either entire or in early stages of fragmentation) included containers, bottles, lids, bottle caps, packaging straps, eel trap cones, oyster spacers, ropes, and fishing nets (Supplementary Table 4). Age and geographical origin evidences were found on some objects, with 50 items having a readable production date: 1 in 1977, 7 in the 1980s, 17 in the 1990s, 24 in the 2000s and 1 from 2010. We also found 386 objects with recognizable words or sentences written in 9 different languages. One third had Japanese inscriptions (115 objects) and another third had Chinese (113 objects). The rest was divided among nine countries (Supplementary Table 5) Furthermore, a country of production was identified on 41 objects (‘made in’ label), manufactured in 12 different countries (see Supplementary Table 5).

### Source, formation and temporal evolution predictions

**Figure 5:**
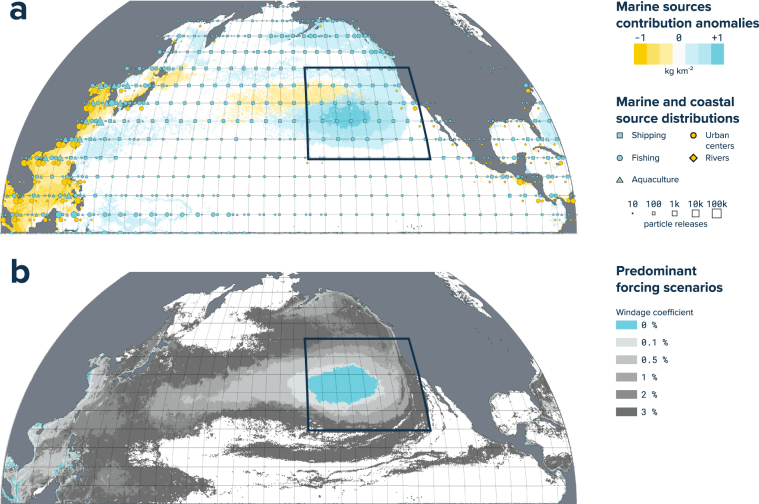
Modelled source and forcing distributions. (**a**) North Pacific distribution of the ocean plastics sources used in this study (blue and orange squares, circles and triangles), and predicted marine source (shipping, fishing and aquaculture) anomalies in relation to the initial distribution of both marine- and land-based sources (coastal urban centres and rivers). (**b**) Predicted North Pacific distribution of dominant forcing scenario. Windage coefficient values correspond to the percentage contributions of wind forcing at 10 m above sea level. Maps were created using QGIS version 2.18.1 (www.qgis.org).

Our global model simulated the release of Lagrangian particles from significant sources of ocean plastic. It predicted that the relative contribution of marine sources (fishing, shipping and aquaculture industries) to the GPGP plastic load was above global average (Fig. 5a).

Model particles were transported under a series of environmental forcing scenarios representing sea surface current (0–10 m), wave-induced Stokes drift, and 0.1%, 0.5%, 1%, 2% and 3% of wind velocities at 10 m above sea surface. The best model representation (*R*^2^ = 0.58, *n* = 277) was established with a windage coefficient of 0%, as this scenario best reproduced the several orders of magnitude differences between concentrations within and around the GPGP region. When considering particles from all forcing scenarios investigated in this study (Fig. 5b), our model predicted that the GPGP is dominated by sea surface current-driven particles, with wind influence increasing as the orbits around the patch become wider. Particles subject to greater atmospheric drag were more likely to escape the GPGP, circling around the North Pacific subtropical gyre if exiting from the south or, entering the North Pacific subpolar gyre near Alaska if leaving from the north. We also noticed that the higher the windage coefficient, the more likely a particle was to encounter landmass.

**Figure 6:**
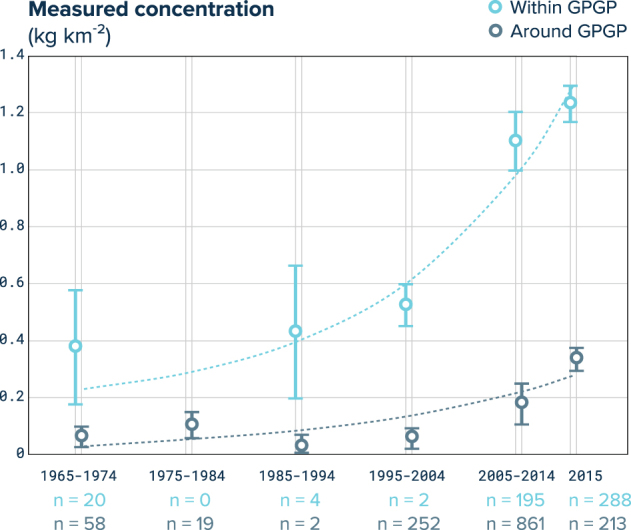
Decadal evolution of microplastic concentration in the GPGP. Mean (circles) and standard error (whiskers) of microplastic mass concentrations measured by surface net tows conducted in different decades, within (light blue) and around (dark grey) the GPGP. Dashed lines are exponential fits to the averages expressed in g km^−2^: *f*(*x*) = exp(a*x) + *b*, with *x* expressed in number of years after 1900, *a* = 0.06121, *b* = 151.3, *R*^2^ = 0.92 for within GPGP and *a* = 0.04903, *b* = −7.138, *R*^2^ = 0.78 for around the GPGP.

Our model resolved the temporal variability of ocean plastic accumulation in the ocean. This allowed us to predict where the GPGP is located at monthly intervals. The GPGP position showed clear inter-annual variations, with latitudinal position of its centre oscillating around 32°N and some frequent temporary displacement southward (26°N to 30°N depending on the forcing scenario). It also demonstrated a clear seasonal variation in the longitudinal position, with its predicted centre oscillating around 145°W and appearing to shift from west to east between boreal winter and summer. Both GPGP latitudinal and longitudinal oscillations intensified with higher atmospheric drag term components. The centre latitude and longitude showed a statistically significant correlation with respectively, the El Niño Southern Oscillation (ENSO^50^; *R* = 0.26, *p* = 0.0011, *n* = 156) and the Pacific Decadal Oscillation (PDO^51^, *R* = 0.71, *p* < 0.001, *n* = 156) indexes. A dynamic GPGP model boundary allowed us to determine whether surface net tows from previous studies^17,21,22,46–49^ were sampling inside or outside the GPGP. Average plastic mass concentration measured by net tows inside the GPGP boundary showed an exponential increase over the last decades, rising from an average 0.4 (±0.2 SE, *n* = 20) kg km^−2^ in the 1970s to 1.23 (±0.06 SE, *n* = 288) kg km^−2^ in 2015 (Fig. 6). Historical samples collected at subtropical latitudes (20°N−45°N) around the GPGP also showed an exponential increase during the same time period but at a slower rate than inside the accumulation zone.

## Discussion

This study provides a detailed quantification and characterization of ocean plastic within a major oceanic plastic pollution hotspot: the GPGP. The sea surface environment of this oceanic region is now dominated by polyethylene (PE) and polypropylene (PP) pieces, substantially outweighing other artificial and natural floating debris. Our aerial survey data, combined with *in-situ* observations from two different trawl devices, supported the development of a comprehensive assessment of all GPGP debris larger than 0.05 cm. Our model estimates that this 1.6 million km^2^ accumulation zone is currently holding around 42k metric tons of megaplastics (e.g. fishing nets, which represented more than 46% of the GPGP load), ~20k metric tons of macroplastics (e.g. crates, eel trap cones, bottles), ~10 k metric tons of mesoplastics (e.g. bottle caps, oyster spacers), and ~6.4 k metric tons of microplastics (e.g. fragments of rigid plastic objects, ropes and fishing nets).

Our plastic mass estimate for the GPGP (~79 k tonnes) was nearly sixteen times higher than a previous study (~4.8 k tonnes) that used net trawl data only^29^ and four times higher than another assessment (~21 k tonnes) that combined net trawl data with vessel-based visual surveys^21^. We suggest that the increase in the estimate is mainly explained by the use of more robust methods for quantifying macro- and megaplastics over larger sea surface areas. For instance, aerial imagery allowed us to more accurately count and measure the size of sighted objects, which undeniably reduced uncertainties in mass estimates when compared to vessel-based visual surveys. Nonetheless, differences between estimates could also be attributed to increasing levels of ocean plastic pollution in the area, and particularly plastic inputs from the 2011 Tohoku tsunami. An estimated 4.5 million tonnes of debris were washed at sea instantly, of which 70% may have sunk rapidly according to Japanese Government^45^. This leaves 1.4 million tonnes of debris that could have been transported at the sea surface over longer distances^44^. The potential tsunami debris contribution to the GPGP is supported by the origin evidences observed on some of the objects collected: Japan was the main production country (34%) of collected plastic objects that had a ‘made in’ label and Japanese was the most common language identified on the objects writings (30%), closely followed by Chinese (29.8%). Considering our estimated global inputs of plastics into the ocean (5.32–19.3 million tonnes y^−1^, see Supplementary Table 6), and assuming tsunami debris have the same fraction of low windage objects than for global sources, our dispersal model suggests the contribution of the 2011 Tohoku tsunami would be around 10–20% of the debris input into the GPGP post 2011.

Despite an increase in the GPGP mass estimate, a great discrepancy between predicted and observed ocean plastic concentrations remains. Considering currently accepted plastic inputs from land- and marine-based sources, our global model predicted millions of tonnes of ocean plastic to be within the GPGP region, while we only found tens of thousands of tonnes. This two-orders of magnitude difference suggests the existence of mechanisms removing most of the plastic mass from the sea surface^16^ and/or fragmenting the plastic into pieces smaller than those quantified here (<0.5 mm^52^). Buoyant plastic represents about 60% of the global plastic demand^10^, thus nearly half of the plastic input into the oceans should sink to the floor soon after release, accumulating in sediments^53^ and underwater canyons^54^. The rest may strand in coastlines^13,55,56^, be ingested by marine life^57^ or removed from the sea surface due to loss of buoyancy through biofouling^58^ or aggregation^59^.

The specific characteristics of the GPGP debris suggest that only certain types of plastic have the capacity to persist at the sea surface for extended periods of time and accumulate in oceanic plastic pollution hotspots. Firstly, the vast majority of the collected GPGP objects were made of PE and PP rigid plastics and bundled fishing nets and ropes. Plastic films however, representing around 37% of PE and PP waste generation^34,60^, were rarely found. We hypothesize that most buoyant plastic with insufficient volume-to-surface ratios such as films may never reach the surface waters of the GPGP as they may rapidly sink to the seafloor due to biofouling^58^ and/or fragment into microscopic pieces that are removed from surface layers^61^.

Secondly, at least half of the collected GPGP plastics was composed of objects from marine based sources, while the relative source amplitudes considered in our model predicted that mass contributions from land-based plastics, even though lower than global average, would still dominate in these offshore environments. This discrepancy could be due to differences in the magnitude of certain removal processes between land-based and marine-based plastics that were not accounted for in our models. We trust that beaching is one of these processes as it may primarily remove plastics that are discarded in coastal environments through wave, tidal and onshore winds transport. Nonetheless, the GPGP dominance of marine-sourced plastics could also be attributed to their purposely engineered durability in the marine environment (e.g. strong and thick-walled nets, traps, ropes, and floats used by marine industries) as well as overestimations of land-based sources and/or underestimations of marine-based sources. In this study, we considered fishing, aquaculture and shipping to be responsible for 28.1% of the global plastic inputs into the oceans, based on coastal clean-up data^62^; however, observations at sea may lead to much higher estimates of plastic loads being lost or discarded at sea. As fishing, shipping and aquaculture intensify globally^42^, it is crucial to better quantify and mitigate this significant source of highly persistent ocean plastic.

Finally, it seems that most plastics accumulating in the GPGP region are hardly transported by winds. Our model predicted that the GPGP is dominated by objects with low or null windage coefficient, and it was the null windage forcing scenario that best represented the GPGP plastic concentrations gradients measured in this study. Furthermore, most objects captured in our trawls (e.g. broken fragments of hard plastic objects, nets and ropes) exhibited no or very little air draft when placed in seawater, and many objects sighted during the aerial expedition seemed fully submerged. Ghostnets, which were the main contributors to the total mass of GPGP plastic, generally have a draft of several metres, and therefore are unlikely to be influenced by wind transport. Our model also suggests that debris items with higher windage are transported over larger areas, with a higher likelihood of beaching, as well as exiting oceanic ‘garbage patches’. For instance, North Pacific particles with 1% wind forcing were spread over a large area around the GPGP that included the Hawaiian archipelago and the North American coastline. The negligible amounts of foam collected within the GPGP, together with the high abundance of foam removed from Alaska during beach clean-ups^63^, and early sightings of highly buoyant debris originated from the 2011 Tohoku tsunami along shorelines (i.e. floating docks, boats and large buoys^44,45^) further suggest that high windage debris may not accumulate within the GPGP region.

It is important to highlight here that our mass estimates are conservative. Most of our sampling effort were conducted inside the GPGP boundary line defined in this study. Although concentrations decreased by orders of magnitude as we moved away from the GPGP centre, when considering the outer GPGP (0.1 kg km^−2^ microplastic contour level, shown in Fig. 3), our model predicted over 100k tonnes of ocean plastics. Moreover, we improved our knowledge on the quantity of plastic contained inside the GPGP over a brief period of time, but we underestimated amounts of higher windage debris that may be passing through the GPGP, while circulating the North Pacific with the subtropical gyre currents. On a monthly average, the concentration of high windage debris within the GPGP may be minimal as it is spread over a larger area than low windage debris. However, when considering debris that passes through the GPGP over a longer period of time, the contribution of high windage debris may be more substantial. Furthermore, some sample biases also made our estimates conservative. Regarding trawl sampling, vessel wake effects were minimized as much as possible, but it is likely that vessel-induced disturbance of the water flow affected the capture efficiency of our nets. Also, both trawls were towed at an angle (so the net moved away from vessel), which means that the width of the sampled area is likely smaller than the net width dimensions used in our area estimations. Finally, megaplastic concentrations estimated from the examination of our aerial mosaics are conservative as some plastics are likely to have been missed by our observers and detection algorithm, or not considered as we only logged features that were clearly recognised as floating plastics.

Historical data from surface net tows (1970–2015) indicate that plastic pollution levels are increasing exponentially inside the GPGP, and at a faster rate than in surrounding waters. While this does not necessarily mean that the GPGP is the final resting place for ocean plastic reaching this region, it provides evidence that the plastic mass inflow is greater than the outflow. The degradation rate of synthetic polymers in the marine environment is poorly understood^64^, but it is known to depend on local environmental conditions, polymer types, shape and coating of objects^10^. The relatively high occurrence of macroplastics with production dates from the 70 s, 80 s, and 90 s compared to more recent debris suggest that specific types of plastic (i.e. with high volume-to-surface ratios and low windage) persist and accumulate in the GPGP region^65^. The mass of plastics floating in the GPGP was mostly distributed in macro- and megaplastics. It is difficult to estimate how long it will take for all the material currently present in the area to degrade in smaller pieces and eventually escape sea surface waters. Based on our modelling results, it seems the bulk mass of material currently present in the GPGP is very unlikely to leave the area and may slowly degrade into increasingly smaller pieces that can eventually either sink to the seafloor^14^, or behave as water tracer due to its microscopic size and low Reynolds number^66^.

Our study provides a comprehensive assessment of the GPGP buoyant plastic loads and characteristics. Nonetheless, a quantification of plastic inputs and outputs into and from the GPGP is required to better assess the residence time of the plastics accumulating in this area. More research effort is needed to quantify ocean plastic sources, transport and loss processes and subsequently implement them in ocean plastic transport models. For instance, no study has recently estimated the global input of fishing gear losses at sea. Furthermore, coastal transport of plastics and its interaction with coastlines worldwide is poorly understood and needs to be implemented in current global models. Levels of plastic pollution in deep water layers and seafloor below the GPGP remain unknown, and could be quantified through sampling. We encourage more sampling throughout the world’s oceans, as well as the systematic monitoring of the GPGP as it is one of the few marine regions with a relatively good historical dataset that allows us to understand long-term trends in oceanic plastic pollution. We also suggest focussing research efforts towards the development of more cost-effective monitoring methods, with better spatio-temporal coverage^67^ and/or capacity to track ocean plastic movements^68^. Air- and space-borne remote sensing technologies may drastically increase our knowledge of ocean plastic transport and certainly represent a great prospect for the future of the ocean plastic research field. Recent advances in commercial satellite imagery for instance may already allow us to identify meter-sized debris items, such as large ghostnets, which are a major contributor to oceanic plastic pollution levels and impacts.

### Data-availability

All datasets associated with this manuscript are available on Figshare^33^.

## Electronic supplementary material

Supplementary Material
