# Supplementary material for: Evidence that the Great Pacific Garbage Patch is rapidly accumulating plastic
Source: Sci Rep. 2018 Mar 22;8:4666. doi: 10.1038/s41598-018-22939-w (PMC5864935; doi:10.1038/s41598-018-22939-w)
Supplement: Supplementary file 1 — Supplementary Material [file 41598_2018_22939_MOESM1_ESM.pdf]

Supplementary Material for 'Evidence that the Great Pacific Garbage Patch is rapidly accumulating plastic'

Lebreton L.[1,2,*], *Slat B.*[1], *Ferrari F.*[1], *Sainte-Rose B.*[1], *Aitken J.*[3], *Marthouse R.*[3], *Hajbane S.*[1], *Cunsolo S.*[1,4], *Schwarz A.*[1], *Levivier A.*[1], *Noble K.*[1,5], *Debeljak P.*[1,6], *Maral H.*[1,7], *Schoeneich-Argent R.*[1,8], *Brambini R.*[1,9], *Reisser J.*[1]

[1] The Ocean Cleanup Foundation, Martinus Nijhofflaan 2, Delft 2624 ES, The Netherlands.

[2] The Modelling House, 66b Upper Wainui Road, Raglan 3297, New Zealand.

[3] Teledyne Optech, Inc., 7225 Stennis Airport Road, Kiln, MS 39556, USA.

[4] School of Civil Engineering and Surveying, Faculty of Technology, University of Portsmouth, Portland Building, Portland Street, Portsmouth, PO1 3AH, UK.

[5] Department of Biology, Marine Biology and Environmental Science, Roger Williams University, 1 Old Ferry Road, Bristol, RI 02809, USA.

[6] Sorbonne Universités, UPMC Univ Paris 06, CNRS, Laboratoire d'Océanographie Microbienne (LOMIC), Observatoire Océanologique, F-66650, Banyuls/mer, France.

[7] Department of Civil, Geo and Environmental Engineering, Technical University Munich, Arcisstraße 21, Munich 80333, Germany.

[8] ICBM-Terramare, Carl von Ossietzky University Oldenburg, Schleusenstr. 1, Wilhelmshaven 26382, Germany.

[9] Civil Engineering Department, Aalborg University, Fredrik Bajers Vei 5, Aalborg 9100, Denmark.

* laurent.lebreton@theoceancleanup.com

SUPPLEMENTARY METHODS

Supplementary Methods 1: Ocean plastic rising velocities and depth-integrated concentrations

The mass and number of ocean plastics captured by surface net tows may not represent the total amount of buoyant plastics present in the area sampled. This is because buoyant plastics can be distributed underwater due to wind-induced vertical mixing. Kukulka et al., 2012 [1] developed a 1-D model that predicts the vertical distribution of buoyant plastics at different sea states. One of the main parameters of this model is the ocean plastic terminal rising velocity (*Wb*), which refers to the constant rising velocity driven by the buoyancy of the object in an undisturbed water column. Previous studies used a constant microplastic *Wb* when applying the model of Kukulka [1] to correct surface plastic measurements for vertical mixing [2] [3] [4]. However, a recent study indicated that this velocity changes with plastic characteristics, such as object shape and size [5]. Here we describe how we measured *Wb*, and used the measurements obtained to estimate the load and number of plastic pieces missed by our surface trawls (sampling depth = 0.15 and 1 m for Manta and Mega trawl, respectively) at different sea states (Beaufort 0 - 5). Terminal rise velocities (*Wb*) of ocean plastics were measured individually, following the method described in Reisser et al., 2015 [4]. We randomly selected 10 - 30 pieces within each of our plastic type/size categories and calculated median *Wb* values. For types 'H' and 'N' within size class 10 - 50 cm, we selected a higher number of pieces (120) to account for the relatively high diversity of objects found within these categories. We measured the length of the 764 tested pieces using calipers (< 5 mm debris) or ruler (> 5 mm debris), then soaked them in water for at least 2 hours prior to the experiment to ensure absence of air bubbles on the pieces' surface. For each piece, the time to rise a certain distance inside a container with water was recorded in triplicates, using a

chronometer. These times were then divided by the distance travelled by the pieces. The mean value of the resulting triplicates was considered the Wb of that piece. As the size of the plastic objects varied largely (0.6 mm - 2.5 m), we used three types of experimental set-ups (Supplementary Figure 1) for measuring Wb:

1. For pieces < 5 mm, we measured the time to rise a distance of 16 cm inside a transparent cylinder (32 cm long, Ø 4 cm) filled with filtered saltwater (salinity 3.5%) and closed airtight with a rubber stopper. The first 16 cm were used for the pieces to stabilise and reach their terminal velocity. Each piece was placed in the cylinder, which was then air-tightened and quickly turned upside down, using a spirit level to adjust its vertical position.
2. For 0.5 - 20 cm pieces, we measured the time to rise a distance of 48 cm inside a transparent tank (100 cm long, Ø 50 cm, water level at 96 cm) filled with filtered salt water (salinity 3.5%). The first 48 cm were used for the pieces to stabilise and reach its terminal velocity. Pieces were released one by one at the bottom of the tank using clamps capable of holding and releasing the pieces as needed.
3. For objects larger than 20 cm, we had to conduct the tests in a freshwater swimming pool (25 m x 15 m) that could accommodate the dimensions of these debris items. For very large objects, we measured the time to rise a distance of 2.45 m in a section of the pool that was 3.6 m deep. The first 1.15 m were used for the stabilization of the object. For the other objects, we measured the time to rise a distance of 98 cm in a section of the pool that was 1.5 m deep. The first 52 cm were used for the objects to stabilise and reach their terminal velocity. The release and observation of objects were made by at least two divers.

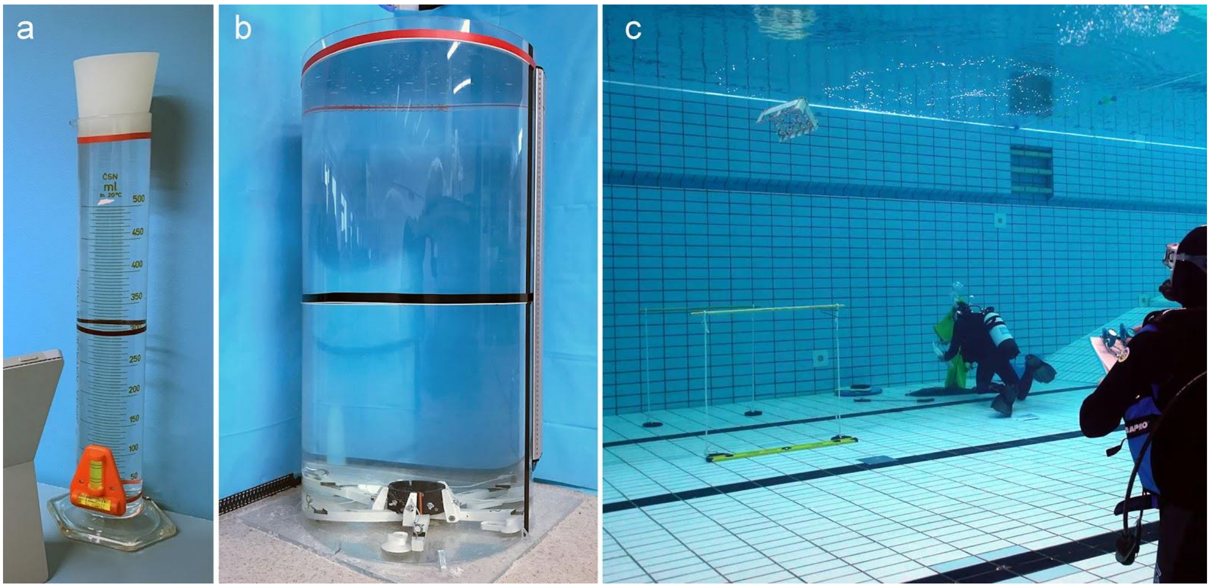

Supplementary Figure 1: **Experimental set-ups used to measure terminal rising velocity (*Wb*) of ocean plastics**. Panel ‘a’ shows the tube used to measure *Wb* of 0.5 - 5 mm plastics, ‘b’ shows the tank used for 0.5 - 20 cm objects, and ‘c’ shows the swimming pool set-up for > 20 cm objects. The lines mid-water (black tapes in ‘a’ and ‘b’, yellow stick in ‘c’) mark the start point for recording the time each particle takes to rise until the water surface.

Since we measured *Wb* of some pieces in freshwater rather than saltwater (n = 237), we had to infer the saltwater *Wb* based on freshwater measurements. To do so, 79 pieces had their *Wb* measured in both fresh (Wb_f) and saltwater (Wb_s) using the Ø 50 cm tank described above. We found a good correlation between the two types of *Wb*, with R^2 = 0.84. As such, we used the resulting regression function ($Wb_s = 0.9755*Wb_f + 1.5478$) to estimate the saltwater *Wb* of those pieces that had their speeds measured in the freshwater pool. The Wb_f of the pieces used in the swimming pool set-up ranged from 1 cm s^{-1} to 35.7 cm s^{-1} (*mean*: 6.6 cm s^{-1}, *SD*: 4.4 cm s^{-1}, n = 237). For these samples, the linear regression function predicted a mean Wb_s 1.21 times (*range*: 1.02 - 2.52) larger than Wb_f.

Measured and estimated saltwater *Wb* values were grouped by the debris type/size categories of this study. Supplementary Figure 2 shows the median, 25th and 75th percentiles of *Wb* for each type/size category. Regardless of plastic type, *Wb* generally increased with object size.

The increase rate is more pronounced for type ‘F’ objects, with median *Wb* ranging from 0.97 cm s^{-1} to 23.95 cm s^{-1}, than for other types (1.31 - 9.10 cm s^{-1} for type ‘H’, 0.47 - 7.43 cm s^{-1} for type ‘N’, and 3.96 - 6.58 cm s^{-1} for type ‘P’). A significant exception was found however for type ‘N’ pieces in the 1.5 - 5 cm size class which showed larger *Wb* than pieces in the 5 - 10 cm range. This may be attributed to the high occurrence of individual rope knots within this size range. These tight knots seem to have a larger volume-to-surface ratio when compared to the elongated and fibrous ropes that dominated the other size classes.

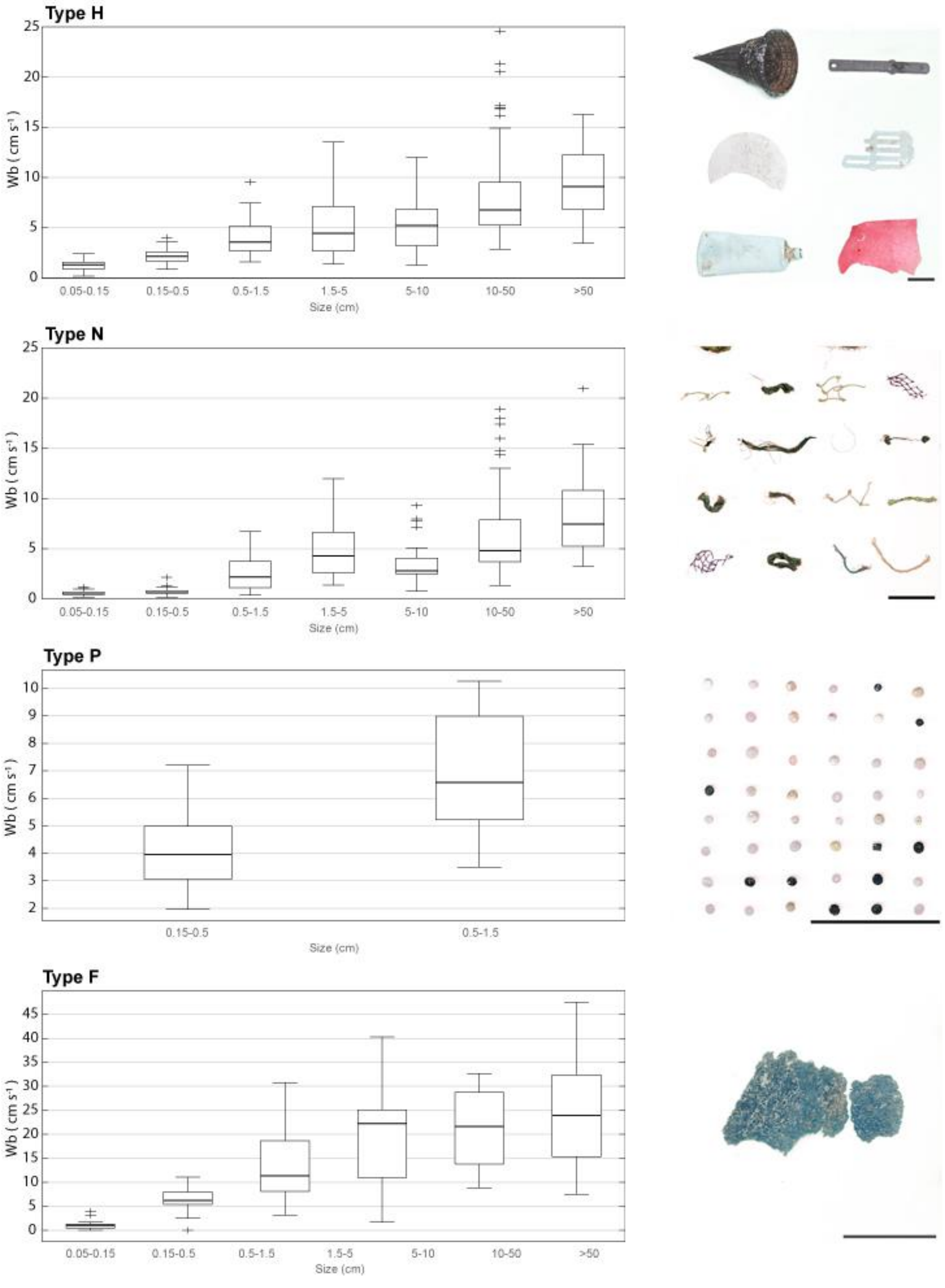

Supplementary Figure 2: Terminal rising velocities (*Wb*) of plastics within different size classes and types. Photographs provide examples of ocean plastic within each type class used in this study. Plastic type H include pieces of hard plastic, plastic sheet and film, type N encompasses plastic lines, ropes and fishing nets, type P are pre-production plastic pellets, and type F are pieces made of foamed material. The black scale bar within each photograph is 5 cm long. Median *Wb* are represented as bold lines, boxes range from 25th to 75th percentiles and

whiskers extend from minimum to maximum values not considering outliers which are plotted as crosses. Two outliers are not shown in the 'Type H' graph: 35.6 and 37.4 cm s^{-1} for the 10 - 50 cm size class.

Our findings on *Wb* were included in the formulation from Kukulka et al., 2012 [1]:

$$C_i = \frac{C_s}{1 - e^{-d w_b A_O^{-1}}}$$

Where: *Cs* is the concentration of a plastic type/size category as measured by the surface net tow (in pieces km^{-2} or kg km^{-2}); *d* is the depth sampled by the net tow, equal to 0.15 m for the Manta trawls and 1 m for the Mega trawls; *Wb* is the median terminal rising velocity (m s^{-1}) of plastic within a plastic type/size category; and A_0 is the near-surface turbulent (eddy) exchange coefficient which was estimated by:

$$A_O = 1.5 u_{*w} k H_s$$

Where: *k* is the von Karman constant, equal to 0.4; u_{*w} is the frictional velocity of water (in m s^{-1}); and H_s is the significant wave height (in m). Following Kukulka et al., 2012 [1], we used the parametric equation to compute H_s:

$$H_s = \frac{0.96}{g} \sigma^{\frac{3}{2}} u_a^{*2}$$

Where: *g* is gravitational constant (in m s^{-2}); σ is the wave age, equal to 35 (assuming a fully developed sea state); and u_{*a} is the air friction velocity (in m s^{-1}). u_{*w} and u_{*a} were derived from:

$$u_w^* = \sqrt{\frac{\rho_a}{\rho_w} C_d U^2} \qquad u_a^* = \sqrt{C_d U^2}$$

Where: ρ_a is the air density; ρ_w the seawater density; C_d the drag coefficient, equal to 1.2 10^{-3}; and *U* is the wind speed during sampling. We considered wind speeds to be equal to 0, 2, 5,

9, 13, and 19 knots for sampling events associated with Beaufort sea states 0, 1, 2, 3, 4, and 5 respectively. To account for uncertainties and variabilities in the wind speeds during sampling and plastic rising velocities, we also estimated ‘minimum’ and ‘maximum’ depth-integrated concentrations for each of the surface concentrations measured by the trawls. The ‘minimum’ depth-integrated concentrations were estimated by using the lowest wind speed of the sea state associated with the field observation (equal to 0, 1, 4, 7, 11, and 17 knots for Beaufort sea states 0, 1, 2, 3, 4, and 5, respectively) and the 75th percentile of the rising velocity measurements observed for each plastic type/size category. The ‘maximum’ depth-integrated concentrations were estimated by using the highest wind speed of the sea state associated with the field observation (equal to 0, 3, 6, 10, 16, and 21 knots for Beaufort sea states 0, 1, 2, 3, 4, and 5 respectively) and the 25th percentile of the rising velocity measurements observed for each plastic type/size category. It is worth noting that some uncertainties lie with the use of this empirical formula as this linear model was originally formulated for microplastics, and here we extrapolate to larger debris sizes. The correction factors used to convert surface plastic concentrations into depth-integrated concentrations are presented in Supplementary Table 7 for Manta trawl and Supplementary Table 8 for Mega trawl.

Supplementary Methods 2: Comparing concentration measurements between Manta and Mega trawls

There is evidence that Manta net tows may underestimate the quantity of large debris items at the sea surface. For instance, while Cózar et al., 2014 [6] estimated that there are 7,000 – 35,000 tonnes of plastics floating in the world's oceans using Manta trawl samples only, Eriksen et al., 2014 [3] estimated that this amount is of at least 250,000 tonnes using Manta trawl samples and data from visual surveys.

On the RV Ocean Starr (largest participating vessel of this study), we concurrently towed four surface trawls at each of its sampling stations (n = 75): two Manta trawls (one from the starboard beam and one from the port beam), alongside two Mega trawls that were towed from the aft of the vessel. These concurrent net tows allowed us to compare 'depth-integrated' plastic concentrations estimations coming from Manta and Mega trawls (Supplementary Figure 3) for the debris size classes sampled by both devices: 1.5 - 5 cm, 5 - 10 cm, 10 - 50 cm, and > 50 cm.

Bland and Altman, 1983 [7] recommended an approach to quantify the agreement between two protocols by plotting the difference in measurements from two methods against the mean of pair measurements. If the measurement methods are in good agreement, the difference should be narrow and centred to 0 in relation to the mean of the measurement pair. We used Bland-Altman plots to assess differences between depth-integrated numerical and mass concentrations coming from Manta and Mega trawls (Supplementary Figure 4).

The Bland-Altman analysis illustrates the zero-inflated distribution of concentrations measured with Manta trawl for objects >5 cm (macroplastics). Data points aligned on the $f(x) = x$ function line represent paired measurements for which Manta trawl failed at collecting debris items while concentrations obtained from the Mega trawl were non-null. Such events clearly start to appear for debris items >5 cm and are obvious for larger sizes. Calculations of

depth-integrated concentrations (blue, green and magenta dots in Supplementary Figure 7) yield more uncertainties for Manta trawl due to a smaller aperture depth, resulting in a wider spread of data points in the $f(x) = -x$ direction. Therefore, we decided to consider the Mega trawl measurement for debris items >5 cm. However, the concentrations measured with the Mega trawl also demonstrated a zero-inflated distribution for debris items >50 cm (megaplastics). Particularly for mass concentration, we calculated a median of 3.1 kg km^{-2} (n = 150) and a maximum of 3,172.3 kg km^{-2} due to the collection of a ghostnet. For the same reason that Manta trawl sampling misrepresents concentrations of debris items >5 cm, we concluded that Mega trawl underestimate concentrations of debris items >50 cm. Thus, we decided to conduct aerial surveys within our study area to sample a larger sea surface area and better quantify this debris size category.

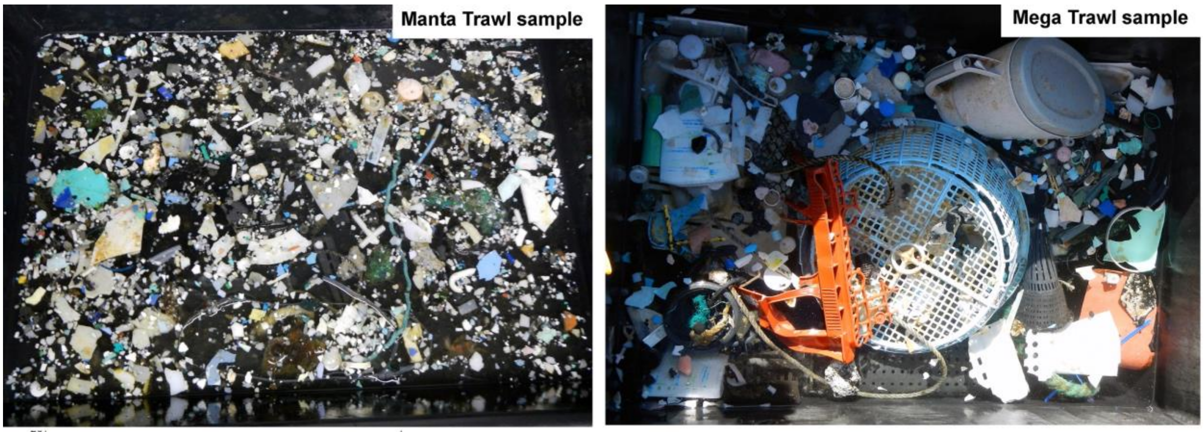

Supplementary Figure 3: **Example of Manta and Mega trawl samples analysed in this study.** Both samples shown here are unprocessed, therefore displaying the whole material collected by the trawl during a net tow within the GPGP area. Note the lack of > 5 cm in the Manta sample, which yielded a zero-inflated distribution for concentrations of > 5 cm ocean plastics for Manta trawl sampling.

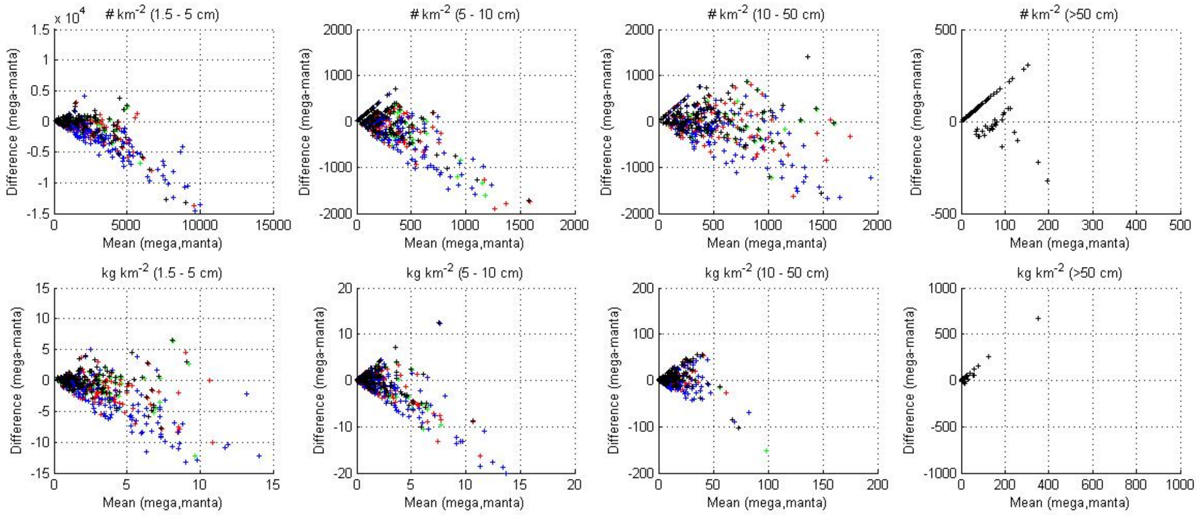

Supplementary Figure 4: Bland-Altman analyses between Manta and Mega trawl sampling. Ocean plastic numerical (top) and mass (bottom) concentration differences between Mega and Manta trawls against respectively mean numerical and mass concentration of the two methods per debris size classes for no depth-integrated correction (black) and minimum (green), median (red) and maximum (blue) depth-integrated concentrations.

Supplementary Methods 3: Estimating mass concentration from aerial imagery

To estimate ocean plastic mass concentrations within each of our 31 RGB mosaics, we had to first estimate the mass of the objects spotted individually. After this we separately summed the mass of the 10 - 50 cm and > 50 cm objects within each mosaic by the area covered.

To estimate the mass of each object spotted on aerial imagery (Supplementary Figure 5), we measured dry weight and 'top-view' length and width of objects collected during trawl surveys that closely resembled the object types and sizes observed in the aerial footage. For footage debris of 'unknown' type, we took a conservative approach and used flat-shaped plastic fragments to estimate their weight. We then developed predictive functions for dry weight, based on the object's type and top-view area. Top-view area for both objects in the laboratory and spotted in the aerial mosaics were calculated from nadir images. For 'bundled nets', we assumed that the top-view area was an ellipse; for 'loose nets', 'ropes', 'containers' and 'unknowns', we multiplied length by width of the object; for 'buoys/lids', we assumed a perfect circle and calculated the surface area from the diameter; and for the only item categorised as 'other' – the life ring – we directly assigned a value by weighing a life ring with same characteristics and dimensions (collected in one of our net tows). To take the nadir photographs of the 'bundled nets' that we collected during the trawl surveys, we had to set-up tanks filled with seawater, so the resulting top-view surface area would better mimic their shape while floating at-sea. All other objects were placed on a table to estimate a conservative top-view surface area while floating at sea. As we found good correlations between top-view surface area and dry weight for different types of objects, we developed a series of predictive functions to estimate debris mass from its top-view area and type (Supplementary Figure 6). We defined a mid-point estimate as well as 95% CI that was used to build our plastic mass concentration confidence intervals. Naturally, the predictive functions for buoys and lids were different. In order to estimate the mass of sighted objects in

the ‘buoy/lid’ category, we weighted the contribution by assuming a 60% chance to the object being a buoy and a 40% chance being a lid. These contributions were calculated from the ratio of buoys and lids (>5 cm) collected in our Mega trawl samples.

Due to the resolution limitation of the aerial images, likely leading to underestimations of 10 - 50 cm debris concentrations (i.e. while mean mass concentrations for 10 - 50 cm debris coming from Mega trawl sampling was equal to 15.1 kg km^{-1}, those coming from the aerial footage were equal to 1.1 kg km^{-1}), we decided to only use the aerial footage concentration estimations for megaplastics (> 50 cm). Furthermore, we did not apply any type of vertical corrections to concentration estimates of debris larger than 50 cm as the estimated Beaufort sea state during aerial surveys was below 3.

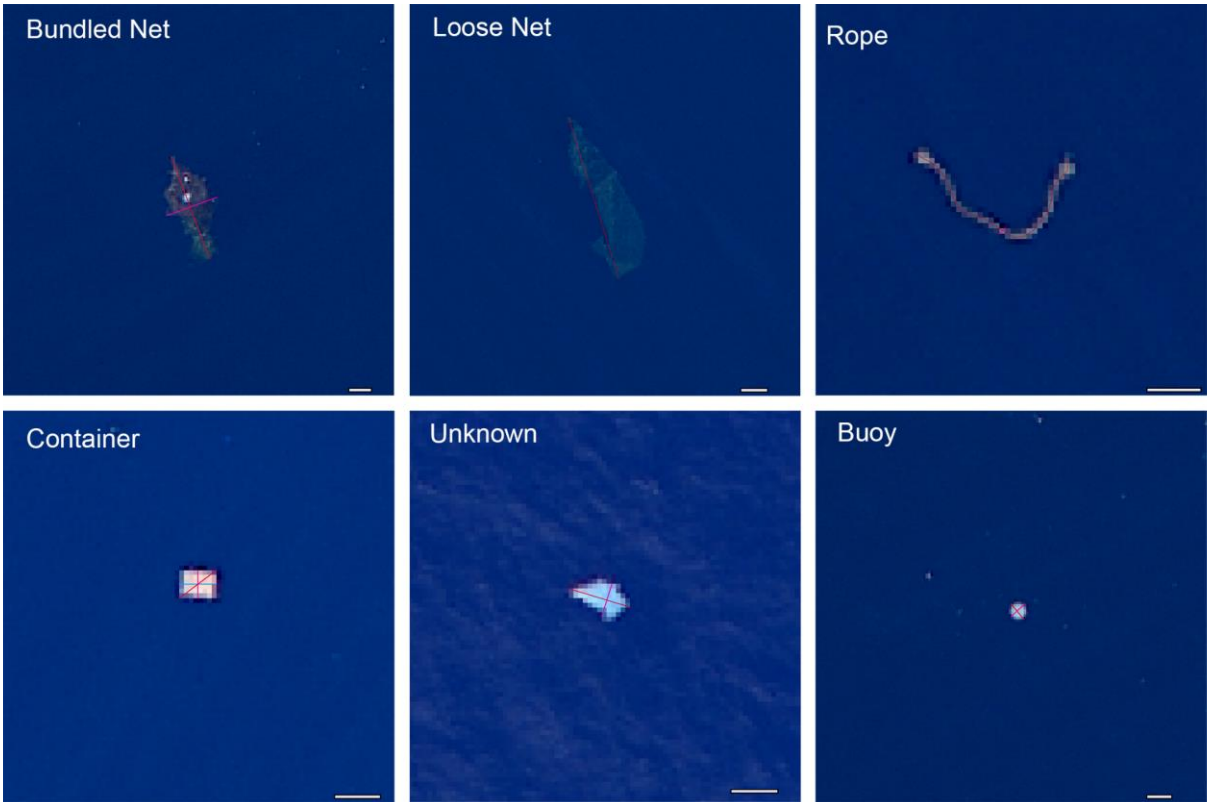

Supplementary Figure 5: Examples of object types observed in the aerial mosaics. Colourful lines show some of the measurements taken. Scale = 1m.

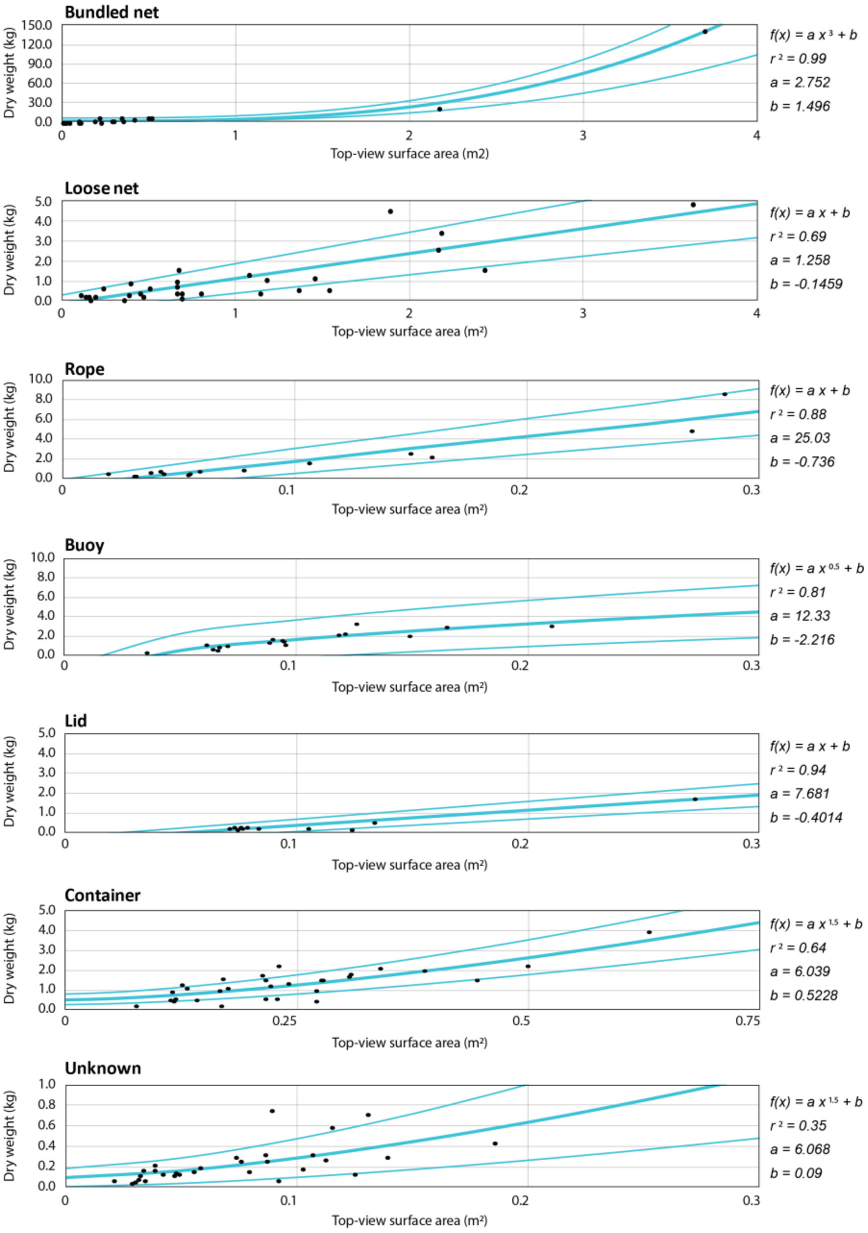

Supplementary Figure 6. Predictive functions for plastic debris mass based on object type and top-view area. Midpoint (bold blue line) and lower/upper from 95 % CI (thin blue lines) are plotted against dry weight x top-view area measurements of objects collected by the trawls of this study (black dots). Predictive functions for the mass of objects used top-view surface area (x) and y is dry weight (y) as input variables. Initial intuitions on power laws was attributed to each object type: we assumed dry weight varies linearly for flat objects (rope, lid and loose net types), to the power of 3/2 (i.e. multiplied by its square roots) for three-dimensional objects (container and unknown fragment types), cubically for bundled nets (representing increase of density from increase in tightness of the nets and aggregation of debris) and by the square root for buoys (i.e. the dry weight varies linearly with the radius of the buoy). The functions, r-square scores and coefficients are shown in the right side of the panels.

Supplementary Methods 4: Modelled sources of ocean plastic

The distribution and rate of particle releases in our ocean plastic dispersal model followed the estimated evolution of relevant ocean plastic source proxies (Supplementary Figure 7): mismanaged plastic waste in coastal areas, plastic inputs from rivers, and losses from fishing, aquaculture, and shipping industries. The five source scenarios represented 4,248,349 particles released from 1993 to 2012. The coastal population (assumed to be within 50 km from the coastline) scenario was based on data from reported litter input estimates from land to the sea for 192 countries around the world [8]. We used country-specific estimates of mismanaged waste generation per inhabitant and per year along with global population density data. We computed the coastal population for individual countries using global 15 x 15-minute population density grids based on IPCC SRES B2 scenario for the years 1990 and 2025 [9] [10] and interpolated using population growth rate based on country-level population and downscaled projections from 1993 to 2012 [11]. For the contribution of inland population (located >50 km from the sea), we used hydrographic model outputs from a global assessment quantifying plastic inputs from rivers [12]. We calculated the temporal evolution of releases from rivers from country-scale population growth data. Plastic waste inputs from the fishing industry were derived from global fishing hotspots. The source distribution was calculated from estimated fishing efforts data per year and per continental fleet [13]. The reported fishing efforts, expressed in kW-days year^{-1}, was computed from catch statistics and fleets location for the period 1990-2006. Bell et al., 2012 [14] reports a breakdown of fishing capacity and effort, per country from 1950 to 2012 using data from the Food and Agriculture Organization of the United Nations (FAO) and other sources. Fishing effort distribution per continental fleets, computed by Watson et al., 2013 [13] was interpolated using the change rate of fishing efforts, calculated by Bell et al., 2012 [14] from 2006 to 2012. The distribution of ocean plastic waste generated by the aquaculture industry was built from aquaculture

production statistics per countries from the United Nation's Food and Agriculture Organisation (FAO [15]). In the FAO's database, aquaculture was categorised by inland aquaculture and mariculture, therefore we considered mariculture production data only. We are not aware of any existing spatial distribution of mariculture infrastructure at global scale. Thus, particles were randomly released inside the spatial overlap between the continental shelf (depth < 200 m, [16]) and each country's exclusive economic zone (EEZ). Finally, the global distribution of plastic sources from the shipping industry was based on gridded shipping frequency [17]. The increase in release rate was proportional to the size of global merchant shipping from 1993 to 2012 [18].

To account for differences in source amplitude, individual particles were attributed a non-dimensional weight based on global input estimates available in the literature for individual sources. For land-based sources (coastal and inland population), we used two global input estimates based on the consideration of mismanaged plastic waste and population density [8] [12]. For marine-based sources (fishing, shipping and aquaculture), we used the average ratio between land- and marine-originated debris found on beaches [19], and incorporated statistical data from European seas on potential marine-based source for collected debris items [20] [21]. To estimate the relative source contribution ranges in percentage of total yearly input, we considered lower (resp. higher) input estimate when all other sources were taken at higher (resp. lower) input estimates. For marine-based sources, the lower and higher input rates were taken from the lower and upper limits of the two global input estimates that were considered for this calculation. The midpoint contributions used to merge our global source scenarios were calculated from the average of lower and upper contribution ranges, then normalized to sum to 100%. Using midpoint relative contribution averages, we calculated that, at global scale, 71.9% of plastic debris originate from land-based sources and 28.1% from direct input at sea (see Supplementary Table 6).

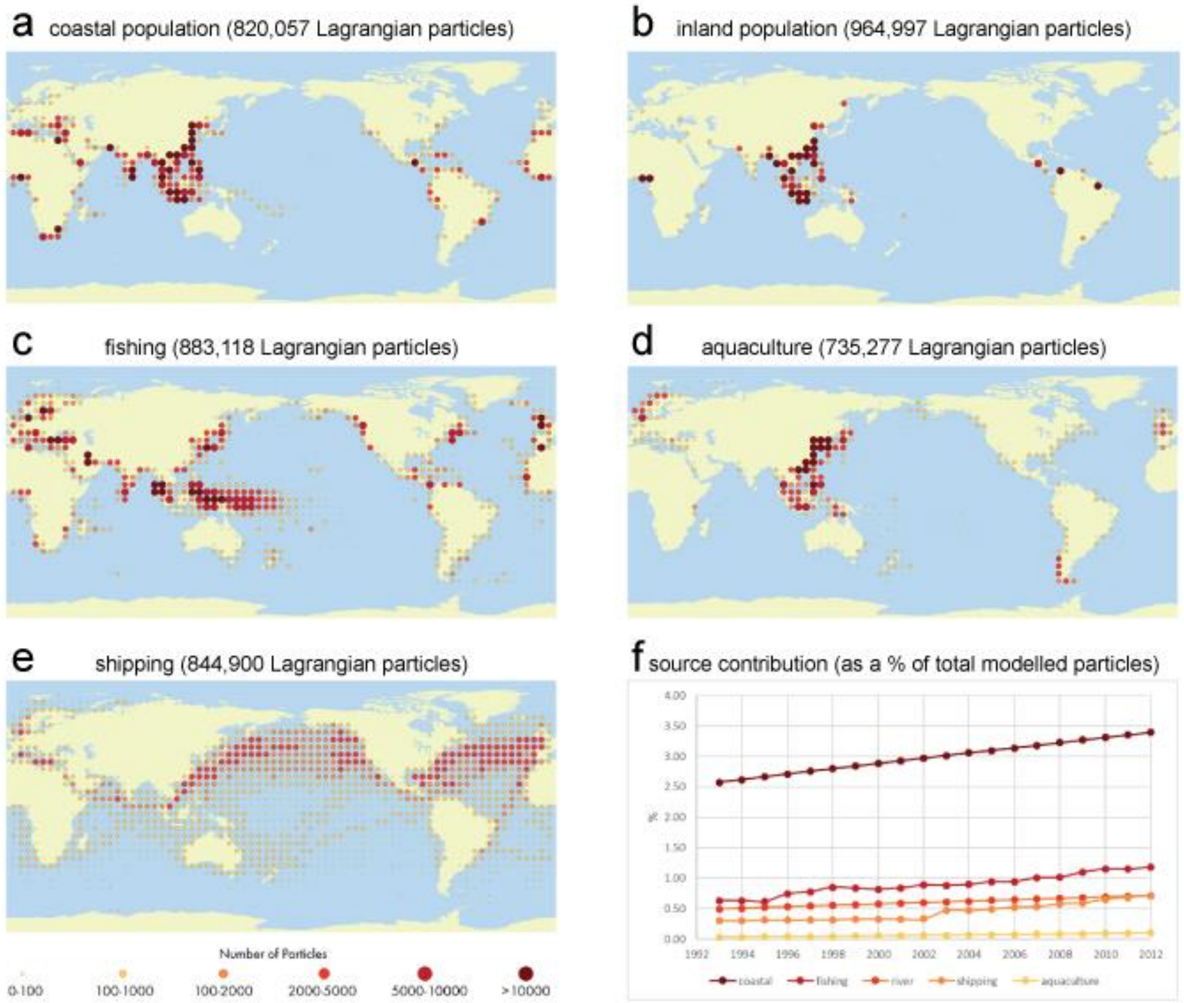

Supplementary Figure 7: **Lagrangian particle source distribution used in the global dispersal model.** Land-based sources (a & b) are derived from mismanaged waste distribution [8] and plastic river inputs [27]. Marine-based sources are representative of fishing effort (c) [13], aquaculture production (d) [15], and shipping frequency (e) [17]. Particles are continuously released from 1993 to 2012 at a yearly rate proportional to respective source proxies (f). Maps were created using QGIS version 2.18.1 (www.qgis.org).

Supplementary Methods 5: Resolving the temporal variability of the GPGP position

We sampled the GPGP between 2015 and 2016 while our ocean plastic model extends from 1993 to 2012. To overcome this issue while accounting for seasonal and inter-annual changes in the GPGP position, we had to search for years in our model reanalysis presenting similar climatic conditions to those experienced during the sampling campaigns. To do so, we first computed the monthly barycentric position of non-dimensional concentration (i.e. mass centre of particle cloud, called GPGP centre thereafter) for the 2000 to 2012 period and all forcing scenarios of this study. Earlier years were not used to allow our model domain to sufficiently accumulate particles, so we could draw reliable contours. We extracted the latitudes and longitudes of the GPGP centre and compared them with two climate indexes (Supplementary Figure 8): The El Niño–Southern Oscillation (ENSO) and the Pacific Decadal Oscillation (PDO). When considering the null windage scenario, the ENSO index showed a statistically significant correlation with both predicted longitude (R = 0.35, p < 0.001, n = 156) and latitude positions (R = 0.26, p = 0.0011, n = 156) of the GPGP centre. The PDO index showed a better correlation (R = 0.71, p < 0.001, n = 156) with the longitude of the GPGP centre than ENSO; however, it did not demonstrate a statistically significant correlation with the latitudinal position (R = 0.13, p = 0.097, n = 156). A positive correlation between ENSO and model-predicted latitudinal position suggests the GPGP is located at higher latitudes during La Niña phases (ENSO positive) and inversely for El Niño phases (ENSO negative). A positive correlation between longitude and PDO suggests that the GPGP is located to the west during PDO negative phase and to the east during PDO positive phase. Our model results exhibited this phenomenon with two noticeable shifts in longitudinal positions in 2003 and 2010, followed by a significant drop in latitudinal position the following months.

Our trawl surveys occurred in July - September 2015, just before a strong La Niña phase (ENSO positive), and our aerial surveys occurred in September - October 2016, just after this La Niña peak (ENSO negative). During our model reanalysis, four significant La Niña events occurred: in 2002 - 2003, 2004 - 2005, 2006 - 2007 and 2009 – 2010 (see Supplementary Figure 8a). The PDO index was also positive for our 2015 trawl surveys and negative for our 2016 aerial surveys, with a PDO peak in between these two expeditions. Of the four La Niña events identified above, only the first two presented similar PDO variations to those experienced during our July 2015- October 2016 sampling period (see Supplementary Figure 8b). As such, we concluded that the GPGP position during our expeditions would be best described with model results for the 2002-2003 and 2004-2005 periods highlighted with grey boxes in both panels of Supplementary Figure 8. Therefore, we used three-monthly averages from July-September 2002 and July-September 2004 to calibrate our model against trawl data and three-monthly averages from September-November 2003 and September-November 2005 to calibrate our model against aerial imagery data.

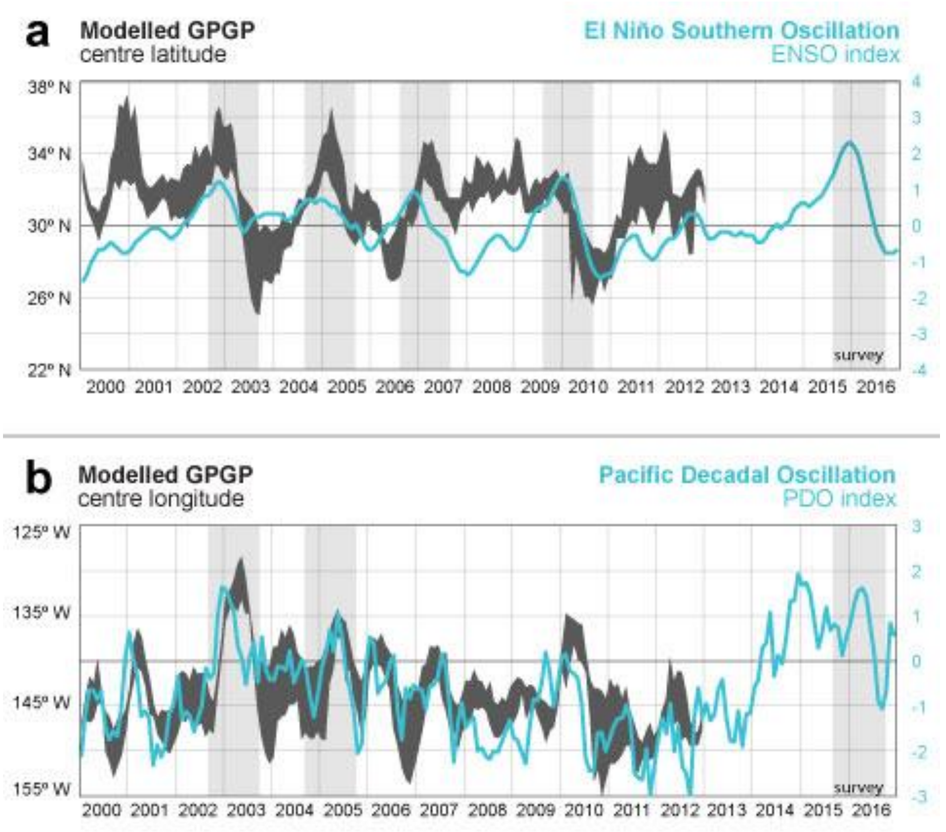

Supplementary Figure 8: Temporal variability of the GPGP centre position. a) Modelled GPGP centre latitude range (thick dark grey line) against the El Niño Southern Oscillation (ENSO) index (blue line). We conducted our surveys before and after a significant la Niña phase (ENSO positive). Four similar events (ENSO positive) starting in 2002, 2004, 2006, 2009 occurred during our model reanalysis period (1999 – 2012). b) Modelled GPGP centre longitude range (thick dark grey line) against the Pacific Decadal Oscillation (PDO) index (blue line). The PDO index transitioned from positive to negative with a likely shift of the GPGP from east to west as predicted for similar events (2002 and 2004) in our model reanalysis.

Supplementary Methods 6: Identifying historical samples collected inside the GPGP

Our ocean plastic model covered the 1993 - 2012 period. It took about six years for released particles to significantly accumulate in the GPGP so that we could draw a clear boundary for this region. Once a boundary was established, we could follow it in time and assess whether a sample was collected inside or outside the GPGP. Samples collected before 1993 were compared against the GPGP position estimated from years in the 1999 - 2012 period that showed similar environmental conditions regarding ENSO and PDO indexes (Supplementary Figure 9). Concentration data reported before 1993 included measurements in 1972, 1973 [22] [23], 1976 [24], and 1985 [25]. The 1972 - 1973 period experienced a similar ENSO event than during our 2015 - 2016 sampling interval: a strong La Niña phase in the first year, followed by a rapid shift to El Niño during the second year. However, the PDO index was negative whereas it was mostly positive during our 2015 – 2016 expeditions. The period 2006 - 2007 and 2009 - 2010 also depicted a strong La Niña to El Niño shift with negative PDO index. Therefore, the average position of the GPGP for these years were compared with samples taken between 1972 and 1973. Similarly, samples collected in 1976 (entering La Niña phase and PDO negative) were compared against the GPGP position predicted respectively for 2002 (entering La Niña phase and PDO negative). As no similar conditions than those experienced in 1985 (two consecutive years of ENSO index near null) were found during our timeseries we conservatively took the 12-year average to estimate the GPGP position for this year. The uncertainties related to detecting microplastics sampling stations occurring inside the GPGP for years previous to 1999 are reflected in the relatively wide confidence intervals presented in Figure 6 of the main manuscript for these decades.

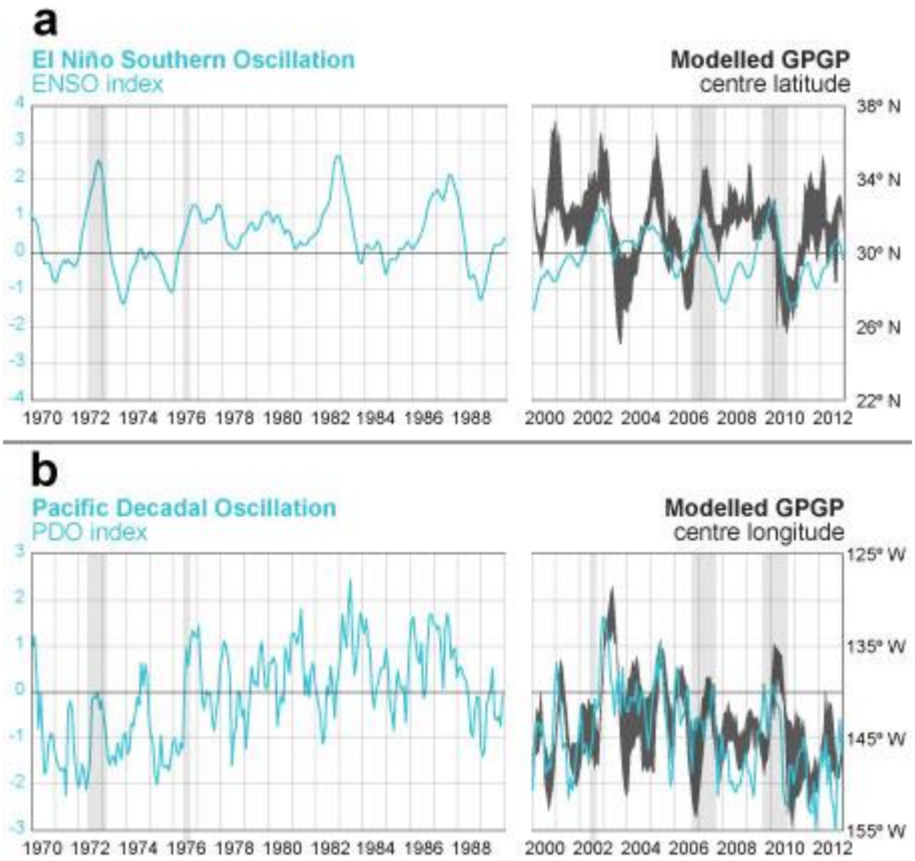

Supplementary Figure 9: **Climate indexes correlation with the GPGP position for the historical dataset analysis.** a) Modelled GPGP centre latitude range (thick dark grey line) against the El Niño Southern Oscillation (ENSO) index (blue line). b) Modelled GPGP centre longitude range (thick grey line) against the Pacific Decadal Oscillation (PDO) index (blue line). We used climatic indexes of periods when net tow data was collected (1972, 1973, and 1976; shown as light grey boxes in the left panels) outside years covered by the model reanalysis period (1999 – 2012) to find similar environmental conditions.

SUPPLEMENTARY TABLES

Size Class (cm)	Type H		Type N		Type P		Type F	
	mass	count	mass	count	mass	count	mass	count
0.05 -0.15	96.17%	94.14%	3.79%	5.82%	0.00%	0.00%	0.03%	0.04%
0.15 - 0.5	92.74%	88.59%	1.42%	7.78%	5.82%	3.55%	0.03%	0.09%
0.5 - 1.5	96.67%	81.36%	3.26%	18.51%	0.02%	0.05%	0.05%	0.08%
1.5 - 5	91.31%	65.12%	8.59%	34.52%	0.00%	0.00%	0.10%	0.35%
5 - 10	94.02%	76.05%	5.94%	23.78%	0.00%	0.00%	0.04%	0.17%
10 - 50	92.12%	61.93%	7.75%	37.91%	0.00%	0.00%	0.13%	0.16%
> 50	7.60%	60.02%	92.40%	39.98%	0.00%	0.00%	0.00%	0.00%

Supplementary Table 1. Mass and count contributions of ocean plastic types to the total mass and count of the size classes considered in this study. Type H include pieces of hard plastic, plastic sheet and film, type N encompasses plastic lines, ropes and fishing nets, type P are pre-production plastic pellets, and type F are pieces made of foamed material.

Sampling Year	Data Reference	Net Type	Reported units	# within (around) NPGP
1972	Goldstein et al. 2012 [22]	Ring net<br>0.5 mm mesh	# m^{-3}<br>mg m^{-3}	8 (23)
1972	Wong et al. 1974 [23]	Neuston<br>0.15 mm mesh	mg m^{-2}	8 (25)
1973	Goldstein et al. 2012 [22]	Neuston<br>0.5 mm	# m^{-2}<br>mg m^{-2}	4 (10)
1976	Shaw & Mapes 1979 [24]	Ring net<br>0.33 mm	mg m^{-2}	0 (19)
1985	Day & Shaw 1987 [25]	Ring net<br>0.33 mm	mg m^{-2}	4 (2)
1999	Moore et al. 2001 [26]	Manta<br>0.33 mm	# m^{-2}<br>mg m^{-2}	2 (8)
2001 - 2004	Law et al. 2014 [2]	Neuston<br>0.33 mm	# km^{-2}	0 (244)
2005 - 2012	Law et al. 2014 [2]	Neuston<br>0.33 mm	# km^{-2}	70 (680)
2007 - 2012	Eriksen et al. 2014 [3]	Manta<br>0.33 mm	# km^{-2}<br>g km^{-2}	29 (114)
2009 - 2011	Goldstein et al. 2012 [22]	Manta<br>0.33 mm	# m^{-3}<br>mg m^{-3}	96 (67)
2015	This Study	Manta<br>0.5 mm	# km^{-2}<br>g km^{-2}	288 (213)

Supplementary Table 2: Historical microplastic concentration dataset. Information related to the plankton net tows considered in our plastic pollution temporal trend analysis for areas within and around the GPGP. White and grey cells show data that were pooled together to represent the following time periods: 1965-1974, 1975-1984, 1985-1994, 1995-2004, 2005-2014, and 2015-present.

Size Class (cm)	Type H	Type N	Type P	Type F
0.05 -0.15	90% PE<br>10% PP	60% PE<br>40% PP	-	10% PE<br>70% PS<br>20% NA
0.15 - 0.5	100% PE	40% PE<br>60% PP	100% PE	50% PE<br>50% PS
0.5 - 1.5	80% PE<br>20% PP	60% PE<br>40% PP	100% PE	30% PE<br>10% PP<br>40% PS<br>10% PVC<br>10% NA
1.5 - 5	70% PE<br>30% PP	80% PE<br>20% PP	-	50% PE<br>40% PS<br>10% PVC
5 - 10	60% PE<br>40% PP	80% PE<br>20% PP	-	30% PE<br>10% PP<br>30% PS<br>10% PVC<br>20% NA
10 - 50	50% PE<br>50% PP	50% PE<br>50% PP	-	70% PE<br>10% PVC<br>20% NA
> 50	60% PE<br>40% PP	80% PE<br>10% PP<br>10% NA	-	-
All	72.9% PE<br>27.1% PP	64.3% PE<br>34.3% PP<br>1.4% NA	100% PE	40% PE<br>3.3% PP<br>38.3% PS<br>6.7% PVC<br>11.7 NA

Supplementary Table 3: Polymer types per ocean plastic type and size within the GPGP. Plastic type H include pieces of hard plastic, plastic sheet and film, type N encompasses plastic lines, ropes and fishing nets, type P are pre-production plastic pellets, and type F are pieces made of foamed material. PE = polyethylene, PP = polypropylene, PS = polystyrene, PVC = polyvinyl chlorine, NA = unknown. Percentages are frequencies of occurrence using 10 pieces per type/size category.

Size Class (cm)	Type H	Type N
0.15 - 0.5	hard plastic fragments (99.6%) film fragments (0.4%)	lines (57.8%), ropes (40.8%), nets (1.4%)
0.5 - 1.5	hard plastic fragments (96.5%), melted plastic (0.8%), film fragments (0.7%), oyster spacers (0.5%), bottle lids (0.4%)	lines (56.1%), ropes (42.7%), nets (1.1%)
1.5 - 5	hard plastic fragments (75.3%), oyster spacers (6.3%), bottle lids (4.8%), container lids (2.9%), melted plastic (2.4%)	ropes (65.5%), lines (33.9%), nets (0.5%)
5 - 10	hard plastic fragments (63.4%), bottles (8.8%), container lids (7.2%), containers (5.6%), oyster spacers (2.5%)	ropes (80.7%), lines (17.6%), nets (1.7%)
10 - 50	hard plastic fragments (43.1%), containers (11.1%), trap cones (10.6%), oyster spacers (10.3%), bottles (6.4%)	ropes (89.5%), lines (6%), nets (4.5%)
> 50	hard plastic fragments (37.5%), packaging straps (20.6%), containers (10.3%), film fragments (10.3%), tubes (9.3%)	ropes (86.7%), nets (12.7%), lines (0.5%),
All	hard plastic fragments (98.2%), film fragments (0.4%), oyster spacers (0.2%), containers (0.2%), bottles (0.1%)	ropes (51.0%), lines (47.1%), nets (1.9%)

* Only the top 5 object types by frequency of occurrence are shown. If less than 5 types are provided, it is because the respective size/type category has less than 5 object types.

Supplementary Table 4: Frequency occurrence (%) of different plastic objects within size classes of the most common ocean plastic types of this study. Plastic type H include pieces of hard plastic, plastic sheet and film, type N encompasses plastic lines, ropes and fishing nets.

Identified languages	Identified country of production
Japanese (115)	Japan (14)
Chinese (113)	Mexico (8)
Korean (65)	Taiwan (5)
English (49)	China (4)
Spanish (33)	Philippines (3)
French (2)	Canada (1)
Vietnamese (2)	Chile (1)
German (1)	Colombia (1)
Portuguese (1)	Germany (1)
Dutch (1)	Italy (1)
Russian (1)	Korea (1)
	Venezuela (1)

Supplementary Table 5: Identified languages and countries of production in plastics collected within the GPGP. The number of objects with recognizable language and/or country of production ('made in' label) is given in parenthesis.

Sources	Global input (in million tonnes year^{-1})	Comments	Midpoint relative source contribution (αs)
Coastal population	4.8 - 12.7	Based on Jambeck et al. 2015 [8] considering input from mismanaged plastic waste produced by coastal population (<50 km from coastline)	**59.8%**
Inland population	0.72 - 1.52	Based on river inputs from Lebreton et al. (2017) [12], considering inputs from inland population (>50 km) and catchments hydrology.	**12.1%**
Fishing	0.29 - 3.5	Based on International Coastal Cleanup (ICC) survey data [19] and European average distributions of marine-based sources in Arcadis 2012 [20]. Assuming 95.4% of emissions in 'Fishing sector' in Eunomia 2016 [21] comes from direct fishing activities (recreational or commercial)	**17.9%**
Aquaculture	0.014 - 0.17	Based on International Coastal Cleanup (ICC) survey data [19] and European average distributions of marine-based sources in Arcadis 2012 [20]. Assuming 4.6% of emissions in 'Fishing sector' in Eunomia 2016 [21] comes from aquaculture.	**1.3%**
Shipping	0.1 - 1.4	Based on International Coastal Cleanup (ICC) survey data [19] and European average distributions of marine-based sources in Arcadis 2012 [20].	**8.9%**

Supplementary Table 6: Global estimates of annual inputs from sources considered in this study.

Midpoint relative source contribution (α_s) are calculated from median and uniformized to have the five source contributions summing to 100 %.

	Beaufort 0 0 knots (0-0)			Beaufort 1 2 knots (1-3)			Beaufort 2 5 knots (4 - 6)			Beaufort 3 9 knots (7 - 10)			Beaufort 4 13 knots (11-16)			Beaufort 5 19 knots (17-21)		
	L	M	H	L	M	H	L	M	H	L	M	H	L	M	H	L	M	H
**Type 'H'**																		
0.05 - 0.15	1	1	1	1	1	1	0.9	1	1	0.4	0.7	1	0.1	0.3	0.5	0.1	0.1	0.2
0.15 - 0.5	1	1	1	1	1	1	1	1	1	0.7	0.9	1	0.2	0.5	0.7	0.1	0.2	0.3
0.5 - 1.5	1	1	1	1	1	1	1	1	1	0.8	1	1	0.3	0.7	0.9	0.2	0.3	0.5
1.5 - 5	1	1	1	1	1	1	1	1	1	0.8	1	1	0.3	0.7	1	0.2	0.3	0.6
5 -10	1	1	1	1	1	1	1	1	1	0.9	1	1	0.4	0.8	1	0.2	0.4	0.6
10 - 50	1	1	1	1	1	1	1	1	1	1	1	1	0.6	0.9	1	0.3	0.5	0.7
> 50 cm	1	1	1	1	1	1	1	1	1	1	1	1	0.7	0.9	1	0.4	0.6	0.8
**Type 'N'**																		
0.05 - 0.15	1	1	1	1	1	1	0.7	0.9	1	0.2	0.3	0.7	0.1	0.1	0.3	0	0	0.1
0.15 - 0.5	1	1	1	1	1	1	0.8	1	1	0.3	0.4	0.8	0.1	0.2	0.3	0	0.1	0.1
0.5 - 1.5	1	1	1	1	1	1	1	1	1	0.5	0.9	1	0.2	0.5	0.8	0.1	0.2	0.4
1.5 - 5	1	1	1	1	1	1	1	1	1	0.8	1	1	0.3	0.7	1	0.2	0.3	0.6
5 -10	1	1	1	1	1	1	1	1	1	0.8	0.9	1	0.3	0.6	0.9	0.2	0.2	0.4
10 - 50	1	1	1	1	1	1	1	1	1	0.9	1	1	0.4	0.8	1	0.2	0.4	0.6
> 50 cm	1	1	1	1	1	1	1	1	1	1	1	1	0.6	0.9	1	0.3	0.5	0.8
**Type 'P'**																		
0.15 - 0.5	1	1	1	1	1	1	1	1	1	0.9	1	1	0.4	0.7	0.9	0.2	0.3	0.5
0.5 - 1.5 cm	1	1	1	1	1	1	1	1	1	1	1	1	0.6	0.9	1	0.3	0.5	0.7
**Type 'F'**																		
0.05 - 0.15	1	1	1	1	1	1	0.7	0.9	1	0.2	0.3	0.7	0.1	0.1	0.3	0	0	0.1
0.15 - 0.5	1	1	1	1	1	1	0.8	1	1	0.3	0.4	0.8	0.1	0.2	0.3	0	0.1	0.1
0.5 - 1.5	1	1	1	1	1	1	1	1	1	0.5	0.9	1	0.2	0.5	0.8	0.1	0.2	0.4
1.5 - 5	1	1	1	1	1	1	1	1	1	0.8	1	1	0.3	0.7	1	0.2	0.3	0.6
5 -10	1	1	1	1	1	1	1	1	1	0.8	0.9	1	0.3	0.6	0.9	0.2	0.2	0.4
10 - 50 cm	1	1	1	1	1	1	1	1	1	0.9	1	1	0.4	0.8	1	0.2	0.4	0.6

Supplementary Table 7: Vertical correction for Manta trawl. Low (L), mid (M) and high (H) vertical correction factor *Cs/*Ci for Manta trawl (sampling depth d = 0.15 m) per sea state and type/size categories of plastic objects. The median correction factor was calculated using the equation from Kukulka et al. 2012 [1] with the median *Wb* of a size/type category and the mean sea surface wind speed relative to a sea state category. The low (resp. high) values were calculated with the 25th (resp. 75th) percentile of *Wb* and the upper (resp. lower) end value of sea surface wind speed relative to a sea state category.

	Beaufort 0 0 knots (0-0)			Beaufort 1 2 knots (1-3)			Beaufort 2 5 knots (4 - 6)			Beaufort 3 9 knots (7 - 10)			Beaufort 4 13 knots (11-16)			Beaufort 5 19 knots (17-21)		
	L	M	H	L	M	H	L	M	H	L	M	H	L	M	H	L	M	H
**Type 'H'**																		
0.05 - 0.15	1	1	1	1	1	1	1	1	1	1	1	1	0.6	0.9	1	0.3	0.6	0.7
0.15 - 0.5	1	1	1	1	1	1	1	1	1	1	1	1	0.8	1	1	0.5	0.7	0.9
0.5 - 1.5	1	1	1	1	1	1	1	1	1	1	1	1	0.9	1	1	0.7	0.9	1
1.5 - 5	1	1	1	1	1	1	1	1	1	1	1	1	0.9	1	1	0.7	0.9	1
5 -10	1	1	1	1	1	1	1	1	1	1	1	1	1	1	1	0.8	1	1
10 - 50	1	1	1	1	1	1	1	1	1	1	1	1	1	1	1	0.9	1	1
> 50 cm	1	1	1	1	1	1	1	1	1	1	1	1	1	1	1	1	1	1
**Type 'N'**																		
0.05 - 0.15	1	1	1	1	1	1	1	1	1	0.8	0.9	1	0.3	0.6	0.9	0.2	0.3	0.4
0.15 - 0.5	1	1	1	1	1	1	1	1	1	0.9	1	1	0.4	0.7	0.9	0.2	0.3	0.5
0.5 - 1.5	1	1	1	1	1	1	1	1	1	1	1	1	0.7	98	1	0.4	0.7	1
1.5 - 5	1	1	1	1	1	1	1	1	1	1	1	1	0.9	1	1	0.7	0.9	1
5 -10	1	1	1	1	1	1	1	1	1	1	1	1	0.9	1	1	0.7	0.8	1
10 - 50	1	1	1	1	1	1	1	1	1	1	1	1	1	1	1	0.8	1	1
> 50 cm	1	1	1	1	1	1	1	1	1	1	1	1	1	1	1	0.9	1	1
**Type 'P'**																		
0.15 - 0.5	1	1	1	1	1	1	1	1	1	1	1	1	1	1	1	0.8	0.9	1
0.5 - 1.5 cm	1	1	1	1	1	1	1	1	1	1	1	1	1	1	1	0.9	1	1
**Type 'F'**																		
0.05 - 0.15	1	1	1	1	1	1	1	1	1	0.8	0.9	1	0.3	0.6	0.9	0.2	0.3	0.4
0.15 - 0.5	1	1	1	1	1	1	1	1	1	0.9	1	1	0.4	0.7	0.9	0.2	0.3	0.5
0.5 - 1.5	1	1	1	1	1	1	1	1	1	1	1	1	0.7	1	1	0.4	0.7	1
1.5 - 5	1	1	1	1	1	1	1	1	1	1	1	1	0.9	1	1	0.7	0.9	1
5 -10	1	1	1	1	1	1	1	1	1	1	1	1	0.9	1	1	0.7	0.8	1
10 - 50 cm	1	1	1	1	1	1	1	1	1	1	1	1	1	1	1	0.8	1	1

Supplementary Table 8: Vertical correction for Mega trawl. Low (L), mid (M) and high (H) vertical correction factor *Cs/C*i for Mega trawl (sampling depth *d* = 1 m) per sea state and type/size categories of plastic objects. The median correction factor was calculated using the equation from Kukulka et al. 2012 [1] with the median *Wb* of a size/type category and the mean sea surface wind speed relative to a sea state category. The low (resp. high) values were calculated with the 25th (resp. 75th) percentile of *Wb* and the upper (resp. lower) end value of sea surface wind speed relative to a sea state category.

SUPPLEMENTARY REFERENCES

1. Kukulka, T., Proskurowski, G., Morét-Ferguson, S., Meyer, D. & Law, K. The effect of wind mixing on the vertical distribution of buoyant plastic debris. *Geophys. Res. Lett.* **39,** 1-6 (2012).

2. Law, K. L. *et al.* Distribution of surface plastic debris in the eastern Pacific Ocean from an 11-year dataset. *Env. Sci. Technol.* **48,** 4732-4738 (2014).

3. Eriksen, M. *et al.* Plastic pollution in the world's oceans: more than 5 trillion plastic pieces weighing over 250,000 tons afloat at sea. *PLoS One* **9**, e111913; 10.1371/journal.pone.0111913 (2014).

4. Reisser, J. *et al.* The vertical distribution of buoyant plastics at sea: an observational study in the North Atlantic Gyre. *Biogeosciences* **12,** 1249-1256 (2015).

5. Kooi, M. *et al.* The effect of particle properties on the depth profile of buoyant plastics in the ocean. *Sci. Rep.* **6,** 33882 (2016).

6. Cózar, A. *et al.* Plastic debris in the open ocean. *PNAS* **111,** 10239-10244 (2014).

7. Bland, J. M. & Altman, D. G. Measuring agreement in method comparison studies. *Stat. Methods Med Res.* **8,** 135-60 (1999).

8. Jambeck, J. R. *et al.* Plastic waste inputs from land into the ocean. *Science* **347,** 768-771 (2015).

9. Yetman, G., Gaffin, S. R. & Xing X. Global 15 x 15 minute grids of the downscaled population based on the SRES B2 scenario, 1990 and 2025. NASA Socioeconomic Data and Applications Center (SEDAC) 10.7927/H4HQ3WTH (2004).

10. Gaffin, S. R., Rosenzweig, C., Xing, X. & Yetman, G. Downscaling and geo-spatial gridding of socio-economic projections from the IPCC special report on emissions scenarios (SRES). *Global Environ. Chang.* **14,** 105-123 (2004).

11. Gaffin, S. R., Xing, X. & Yetman, G. Country-level GDP and downscaled projections based on the SRES A1, A2, B1, and B2 marker scenarios, 1990-2100. NASA Socioeconomic Data and Applications Center (SEDAC) 10.7927/H4XW4GQ1 (2002).

12. Lebreton, L. *et al.* River plastic emissions to the world's oceans. *Nat. Commun.* **8,** 15611; 10.1038/ncomms15611 (2017).

13. Watson, R.A. *et al.* Global marine yield halved as fishing intensity redoubles. *Fish Fish.* **14,** 493-503 (2013).

14. Bell, J. D., Watson, R. A. & Ye, Y. Global fishing capacity and fishing effort from 1950 to 2012. *Fish Fish.* **18,** 792-793 (2016).

15. FAO. AQUASTAT main database. Retrieved on 24/04/2016 at www.fao.org/nr/aquastat/ (2016).

16. GEBCO. The GEBCO_2014 Grid (version 20150318, BODC). Retrieved on 24/04/2016 at https://www.gebco.net/data_and_products/gridded_bathymetry_data/ (2014).

17. Halpern, B. S. *et al.* A global map of human impact on marine ecosystems. *Science* **319,** 948-952 (2008).

18. UNCTAD. Merchant fleet by flag of registration and by type of ship, annual, 1980-2016. Retrieved on 11/04/2016 at http://unctadstat.unctad.org (2016).

19. International Coastal Cleanup. Tracking trash - 25 years of action for the ocean. Preprint at http://act.oceanconservancy.org/pdf/Marine_Debris_2011_Report_OC.pdf (2011).

20. Arcadis. Economic assessment of policy measures for the implementation of the marine strategy framework directive. Preprint at http://ec.europa.eu/environment/enveco/water/pdf/report.pdf (2013).

21. Eunomia. Study to support the development of measures to combat a range of marine litter sources. Preprint at http://ec.europa.eu/environment/marine/good-environmental-status/descriptor-10/pdf/MSFD%20Measures%20to%20Combat%20Marine%20Litter.pdf (2016).

22. Goldstein, M. C., Rosenberg, M. & Cheng, L. Increased oceanic microplastic debris enhances oviposition in an endemic pelagic insect. *Biol. Lett.* **8,** 817-820 (2012).

23. Wong, C. S., Green, D. R. & Cretney, W. J. Quantitative tar and plastic waste distributions in the Pacific Ocean. *Nature* **247,** 30-32 (1974).

24. Shaw, D. G. & Mapes G. A. Surface circulation and the distribution of pelagic tar and plastic. *Mar. Pollut. Bull.* **10,** 160-162 (1979).

25. Day, R. H. & Shaw, D. G. Patterns in the abundance of pelagic plastic and tar in the North Pacific Ocean, 1976–1985. *Mar. Pollut. Bull.* **18,** 311-316 (1987).

26. Moore, C. J., Moore, S. L., Leecaster, M. K. & Weisberg, S. B. A comparison of plastic and plankton in the North Pacific central gyre. *Mar. Pollut. Bull.* **42,** 1297-1300 (2001).
